# A Tale of Usurpation and Subversion: SUMO-Dependent Integrity of Promyelocytic Leukemia Nuclear Bodies at the Crossroad of Infection and Immunity

**DOI:** 10.3389/fcell.2021.696234

**Published:** 2021-08-27

**Authors:** Upayan Patra, Stefan Müller

**Affiliations:** Institute of Biochemistry II, Faculty of Medicine, Goethe University, Frankfurt, Germany

**Keywords:** promyelocytic leukemia nuclear bodies, small ubiquitin-related modifier, viruses, bacteria, intrinsic and innate immune response

## Abstract

Promyelocytic leukemia nuclear bodies (PML NBs) are multi-protein assemblies representing distinct sub-nuclear structures. As phase-separated molecular condensates, PML NBs exhibit liquid droplet-like consistency. A key organizer of the assembly and dynamics of PML NBs is the ubiquitin-like SUMO modification system. SUMO is covalently attached to PML and other core components of PML NBs thereby exhibiting a glue-like function by providing multivalent interactions with proteins containing SUMO interacting motifs (SIMs). PML NBs serve as the catalytic center for nuclear SUMOylation and SUMO-SIM interactions are essential for protein assembly within these structures. Importantly, however, formation of SUMO chains on PML and other PML NB-associated proteins triggers ubiquitylation and proteasomal degradation which coincide with disruption of these nuclear condensates. To date, a plethora of nuclear activities such as transcriptional and post-transcriptional regulation of gene expression, apoptosis, senescence, cell cycle control, DNA damage response, and DNA replication have been associated with PML NBs. Not surprisingly, therefore, SUMO-dependent PML NB integrity has been implicated in regulating many physiological processes including tumor suppression, metabolism, drug-resistance, development, cellular stemness, and anti-pathogen immune response. The interplay between PML NBs and viral infection is multifaceted. As a part of the cellular antiviral defense strategy, PML NB components are crucial restriction factors for many viruses and a mutual positive correlation has been found to exist between PML NBs and the interferon response. Viruses, in turn, have developed counterstrategies for disarming PML NB associated immune defense measures. On the other end of the spectrum, certain viruses are known to usurp specific PML NB components for successful replication and disruption of these sub-nuclear foci has recently been linked to the stimulation rather than curtailment of antiviral gene repertoire. Importantly, the ability of invading virions to manipulate the host SUMO modification machinery is essential for this interplay between PML NB integrity and viruses. Moreover, compelling evidence is emerging in favor of bacterial pathogens to negotiate with the SUMO system thereby modulating PML NB-directed intrinsic and innate immunity. In the current context, we will present an updated account of the dynamic intricacies between cellular PML NBs as the nuclear SUMO modification hotspots and immune regulatory mechanisms in response to viral and bacterial pathogens.

## Introduction

Promyelocytic leukemia nuclear bodies (PML NBs) are ubiquitous, multi-molecular protein condensates associated with the nuclear matrix (Lallemand-Breitenbach and de Thé, 2018; Corpet et al., 2020). By immunofluorescence microscopy, PML NBs are detected as punctate structures in inter-chromatin spaces. Number, size, and morphology of PML NBs vary depending on cell type and cell cycle status as well as on physiological or pathological stimuli. The composition of PML NBs is heterogeneous and most of the constituents remain in dynamic equilibrium between the soluble nucleoplasmic and the insoluble matrix-associated fraction (Lallemand-Breitenbach and de Thé, 2018; Corpet et al., 2020). Core to the compositional heterogeneity and functional maturity of PML NBs is a specific post translational modification (PTM) with small ubiquitin-related modifier (SUMO) proteins (Cheng and Kao, 2013; Sahin et al., 2014a). PML NBs have widely been regarded as the nuclear niche for protein SUMOylation and most of the constitutive and transient PML NB components are covalently or non-covalently associated with SUMO. PML NBs exert crucial modulatory roles in various activities including nuclear protein quality control, control of gene expression, apoptotic cell death and senescence, genome replication and genomic integrity as well as interferon (IFN) response and antiviral defense (Everett and Chelbi-Alix, 2007; Hsu and Kao, 2018; Lallemand-Breitenbach and de Thé, 2018). Accordingly, the impact of SUMO-dependent regulation of PML NBs on cellular physiology is multifaceted. One of these facets is the regulation of intrinsic and innate immune defense in response to invading pathogens. Many reports have enunciated crucial interplays between PML NBs as the hubs of cellular intrinsic immune defenses and viral countermeasures to establish infection and to ensure perpetuation (Geoffroy and Chelbi-Alix, 2011; Scherer and Stamminger, 2016). Emerging evidence, however, is accumulating in favor of viruses to selectively counteract certain components of PML NBs while usurping others (Guion et al., 2019; Guion and Sapp, 2020). In addition to viruses, certain bacterial pathogens also target PML NB components (Ribet and Cossart, 2018). Notably, the interplay between PML NBs and pathogens might involve the host cellular SUMO machinery as an intermediate or might be independent of SUMO-dependent regulations (Everett et al., 2013b; Scherer and Stamminger, 2016; Lowrey et al., 2017; Wilson, 2017; El Motiam et al., 2020). Here, we present an updated overview of how PML NBs can serve as the niches for mounting intrinsic and innate immune defenses against different pathogens and, in turn, how they are antagonized by the pathogens. We have also highlighted how SUMO-dependent regulations can dictate the outcome of such interplays.

## Structural Integrity of PML NBs and Sumoylation

**FIGURE 1:**
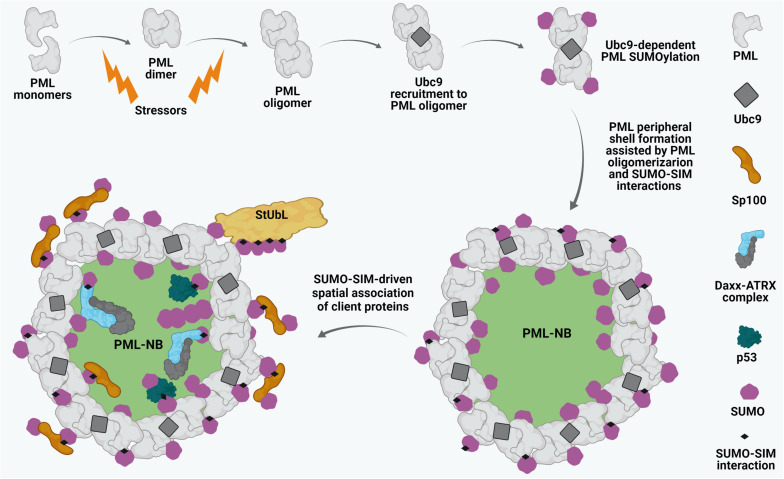
Biogenesis of PML NBs. Oxidative stressors induce PML dimerization and oligomerization through covalent disulphide linkage and intermolecular RBCC motif-driven non-covalent interactions. Subsequently, the SUMO E2 conjugating enzyme Ubc9 is recruited to oligomerized PML and drives PML SUMOylation which further leads to stabilized PML NB shell formation. Sp100 resides along with SUMO1 at the peripheral shell. SUMO-SUMO interacting motif (SIM) affinity also enables recruitment of different client proteins such as Daxx, ATRX, p53 onto mature PML NBs. Ubc9-dependent SUMOylation of the clients may in turn regulate their dynamic interactions with the PML NBs. Poly-SUMO-primed PML might also interact with SUMO targeted ubiquitin ligases (StUbLs), suggesting crucial dynamicity of these matrix-associated sub-nuclear foci.

Promyelocytic leukemia protein constitutes the primary structural scaffold for PML NBs (Ishov et al., 1999; Lang et al., 2010). The biomedical interest in PML NBs stems from the initial observation that their structural integrity is lost in acute promyelocytic leukemia (APL). Disruption of NBs in APL is caused by expression of the oncogenic fusion protein PML-RARα (PML-retinoic acid receptor alpha) resulting from the t(15; 17) chromosomal translocation (de Thé et al., 1991; Kakizuka et al., 1991; Dyck et al., 1994; Weis et al., 1994). Similarly, experimental ablation of PML results in complete disassembly of PML NBs (Ishov et al., 1999; Zhong et al., 2000). Due to alternative splicing, PML has 7 isoforms (I-VII). All the isoforms possess a conserved N-terminal region which is comprised of a RING finger domain, two B- boxes (B1, B2), and an α-helical coiled-coil domain, defining PML (also known as TRIM19) as a member of the tripartite RBCC/TRIM motif family. With the exception of isoform VII, which lacks a nuclear localization signal (NLS), all other isoforms (PML I-VI) exhibit a predominantly nuclear localization and can take part in forming PML NBs (de Thé et al., 2012; Nisole et al., 2013). The biogenesis of PML NBs is a dynamic biphasic process. The initial nucleation phase includes the polymerization of PML proteins into a peripheral shell. Covalent disulfide linkages between oxidized PML monomers as well as intermolecular non-covalent interactions between RBCC domains of PML proteins have been proposed to contribute to this event (Figure 1; Lallemand-Breitenbach et al., 2001; Jeanne et al., 2010; Sahin et al., 2014c). The subsequent maturation phase of PML NB biogenesis involves the recruitment of a heterogeneous assembly of proteins (client proteins) within the inner core of the scaffold in either a constitutive or a stimulus-dependent fashion (Figure 1; Lallemand-Breitenbach and de Thé, 2018). Importantly, the recruitment and release of these inner core clients are primarily dictated by SUMO-dependent protein-protein interactions.

**FIGURE 2:**
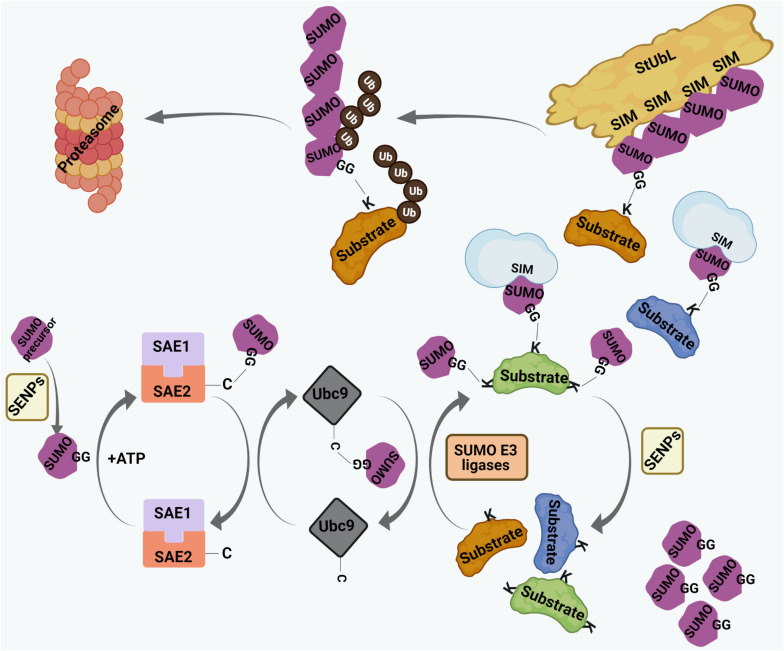
The process of SUMO conjugation and deconjugation. SUMO precursors are proteolytically cleaved by Sentrin-specific proteases (SENPs) to yield conjugation-competent SUMO molecules with terminal di-glycine (GG) motif. SUMO conjugation involves sequential involvement of 3 enzymes – the SUMO E1 activating enzyme (SAE1/SAE2 dimer in human), the SUMO E2 conjugating enzyme (Ubc9 in human), and a limited subset of SUMO E3 ligases. SUMO makes transient, intermediate thioester linkage to cysteine (C) residues of SAE2 and Ubc9 before being finally transferred on to the lysine (K) residues of substrates. The terminally exposed GG of each SUMO forms a covalent isopeptide bond with the ε-amino group on target K residue. Targets can either be monoSUMOylated at single K residue, multi-mono SUMOylated at many K residues or poly-SUMOylated forming SUMO chains. SUMO residues on SUMO-conjugated proteins interact non-covalently with proteins having SUMO interacting motif (SIM). SUMO conjugation can be reversed by SENPs where SUMO residues are cleaved off the substrates. SUMO chains are also targeted by a specific set of E3 ubiquitin ligases, called SUMO targeted ubiquitin ligases (StUbLs), having multiple tandemly arranged SIMs. StUbLs can induce proteolytic/non-proteolytic ubiquitylation of poly-SUMO-primed substrates; the former leads to proteasomal degradation of the substrates.

Small ubiquitin-related modifier proteins are covalently conjugated to their target proteins at lysine (K) residues (Figure 2). In human, three main conjugation-competent SUMO paralogs (SUMO1, SUMO2, SUMO3) are expressed. SUMO2 and SUMO3 differ by 3 amino acids whereas SUMO1 has 50% sequence identity with SUMO2/3 (Saitoh and Hinchey, 2000; Flotho and Melchior, 2013). There is also a SUMO4 paralog which has restricted tissue distribution and cannot be conjugated to target proteins (Bohren et al., 2004; Owerbach et al., 2005). The recently identified SUMO5 is annotated as a pseudogene by the HUGO Gene Nomenclature Committee (HGNC) and therefore it remains under debate whether SUMO5 is expressed at the protein level (Liang et al., 2016). The conjugation of SUMO is a three-step process with sequential involvement of a SUMO E1 activating enzyme (SAE1/SAE2 dimer in human), an E2 conjugating enzyme (Ubc9 in human), and a limited subset of E3 ligases (such as RanBP2, PIAS family members, or ZNF451) (Figure 2). Depending on the type of conjugation, a target can be either mono-SUMOylated at a single K residue, multi-mono SUMOylated at several K residues, or may undergo polySUMOylation by forming SUMO chains on internal K residues within SUMO (Figure 2; Ulrich, 2008; Jansen and Vertegaal, 2021). SUMO-conjugated proteins can make non-covalent interactions with proteins harboring distinct SUMO interacting motifs (SIMs) (Figure 2). The best characterized SIM is composed of a hydrophobic core flanked by acidic amino acids or serine/threonine (S/T) residues. SUMO-SIM affinity is widely used to regulate the dynamics of protein-protein interactions in large multi-molecular complexes, including PML NBs (Ullmann et al., 2012; Raman et al., 2013; Husnjak et al., 2016). Of importance, polymeric SUMO2/3 chains can trigger a specific signaling process known as the SUMO-targeted ubiquitin ligase (StUbL) pathway, where distinct E3 ubiquitin ligases, termed StUbLs, interact with SUMO2/3 multimers/polymers through their tandemly repeated SIM regions (Figure 2). The two characterized mammalian StUbLs, RNF4 and RNF111, trigger proteolytic or non-proteolytic ubiquitylation of poly-SUMO primed substrates (Lallemand-Breitenbach et al., 2008; Tatham et al., 2008; Geoffroy and Hay, 2009; Sriramachandran and Dohmen, 2014; Keiten-Schmitz et al., 2020). SUMO-modifications are reversed by SUMO specific isopeptidases which catalyze cleavage of covalently attached SUMO moieties or SUMO chains from substrates (Figure 2). The best-studied deconjugases belong to the SENP (Sentrin protease) family (Figure 2). Each SENP family member (SENP1-3, SENP5-7 in human) is responsible for deconjugating SUMO residues from a subset of SUMO substrates (Nayak and Müller, 2014; Kunz et al., 2018), thereby providing plasticity within the SUMO system. Dysregulation of the finely tuned balance between SUMO-conjugation and deconjugation can lead to many pathological conditions.

Interestingly, SUMO-SIM interactions are critical for the assembly of mature PML NBs (Figure 1). A large number of cell-biological studies have demonstrated that SUMO-SIM dependent protein-protein interactions within PML NBs form the scaffold of these structures. More recent *in vitro* biophysical and biochemical data now underscore this concept by demonstrating that the accumulation of polySUMO-polySIM polymers results in formation of droplet-like structures and also allows interactions with SUMO/SIM-containing clients depending upon their valency (Banani et al., 2016). Moreover, within cells, these SUMO-SIM polymers were shown to form condensates especially at telomeric regions and to regulate partitioning of client proteins (Banani et al., 2016; Min et al., 2019; Zhang et al., 2020). These results advocate for a SUMO-SIM-dependent liquid-liquid phase separation (LLPS) model for PML NB maturation after PML-driven peripheral shell formation.

Promyelocytic leukemia contains three major SUMO conjugation sites at K65, K160 and K490, which are targeted by SUMO1 and SUMO2/3 (Kamitani et al., 1998). PML is polySUMOylated by SUMO2/3 primarily at K160 residue (Tatham et al., 2008). Further, PML isoforms I-V also possess a phospho-regulated SIM (amino acid 556–562) (Stehmeier and Muller, 2009). PML recruits Ubc9 to PML NBs through its RING finger domain, thereby mediating SUMOylation of PML and enabling SUMO-SIM-dependent PML oligomerization (Figure 1; Shen et al., 2006; Sahin et al., 2014c). PML SUMOylation is also important for recruiting additional client proteins to the inner core of mature PML NBs (Figure 1; Shen et al., 2006). Close to 200 client proteins have now been reported to be associated with PML NBs either constitutively or transiently (Van Damme et al., 2010). A subset of proteins associated with PML NBs functions as chromatin and/or transcriptional regulators. One example is Sp100, which was one of the first proteins identified to be localized to PML NBs (Szostecki et al., 1990; Sternsdorf et al., 1995). In human, at least four alternatively spliced isoforms of Sp100-Sp100A, Sp100B, Sp100C, and Sp100HMG-varying at their C-terminal, are present. Sp100B, Sp100C, and Sp100HMG harbor SAND DNA binding domain and are involved with chromatin-dependent transcriptional regulation (Seeler et al., 2001; Negorev et al., 2006, 2009). All Sp100 forms reside in the peripheral shell of PML NBs (Lang et al., 2010) and are extensively SUMOylated (Figure 1). Notably, however, recruitment of Sp100 to PML NBs does not require covalent SUMO conjugation to Sp100, but involves binding of a SIM in Sp100 to SUMO-conjugated PML (Figure 1; Sternsdorf et al., 1997, 1999). Interestingly, lack of Sp100 expression also affects PML expression, underlining the importance of Sp100 in maintaining PML NB integrity (Negorev et al., 2009). Another component of the PML NB core is the chromatin remodeling factor death domain-associated protein (Daxx) which forms a histone chaperone complex with a partner protein alpha-thalassemia retardation syndrome x-linked (ATRX) (Figure 1) and regulates histone H3.3 depositions on DNA in replication-independent manner, thereby modulating transcriptional repression (Lewis et al., 2010). Similar to what is observed for Sp100, recruitment of Daxx to PML NBs depends on the interaction of Daxx SIM with SUMO-conjugated PML (Figure 1; Lin et al., 2006). Accordingly, the SUMOylation status of PML regulates Daxx recruitment to and release from PML NBs (Lehembre et al., 2001; Kawai et al., 2003; Nefkens et al., 2003). SUMO paralogs and SUMO E3 ligases, such as members of the protein inhibitor of activated STAT (PIAS) family, are also compartmentalized in PML NBs (Lang et al., 2010; Rabellino et al., 2012; Brown et al., 2016). Moreover, p53 and a number of p53 regulatory proteins associate with PML NBs, suggesting the significance of these subnuclear foci to transduce anti-proliferative and pro-apoptotic signals (Figure 1; Bernardi et al., 2008).

Certain stimuli such as arsenic trioxide (As_2_O_3_) shift the equilibrium of intra-nuclear PML distribution toward the insoluble matrix-associated fraction leading to increased size and number of PML NBs. Importantly, phosphorylation and SUMOylation of PML have been shown to be involved in this dynamic shift in the equilibrium as As_2_O_3_ treatment resulted in an enrichment of the phosphorylated pool of PML leading to its recruitment to PML NBs and an increase of PML SUMOylation (Lallemand-Breitenbach et al., 2001; Hayakawa and Privalsky, 2004). As an alternative model, direct binding of As_2_O_3_ to PML’s RING domain has been proposed to induce its dimerization and SUMOylation (Zhang et al., 2010). Interestingly, As_2_O_3_ also induces SUMO chain formation on PML and the oncogenic fusion protein PML-RARα, thereby activating RNF4-dependent ubiquitylation and proteasomal degradation of PML (Figure 1) and PML-RARα (Lallemand-Breitenbach et al., 2008; Tatham et al., 2008).

## Interplay Between PML NBs and Interferon Signaling

Promyelocytic leukemia NBs regulate the IFN-dependent innate immune signaling which constitutes the primary defense arm of the host cells upon encountering a pathogenic entity. The cellular IFN response is initiated upon sensing of pathogen associated molecular patterns (PAMPs) by host cellular pattern recognition receptors (PRRs). The subsequent signal amplification step culminates in the transcriptional upregulation of many IFN stimulated genes (ISGs) through Janus kinase (JAK)-Signal transducer and activator of transcription (STAT) signaling pathway (Schneider et al., 2014). Importantly, PML expression is strongly induced in response to IFNs [type I (IFNα, IFNβ) as well as type II (IFNγ)] (Chelbi-Alix et al., 1995). Consistently, the size and number of PML NBs increase upon IFN stimulation (Chelbi-Alix et al., 1995). The promoter region of the *PML* gene contains both an IFN-stimulated response element (ISRE) and a gamma activated site (GAS) consensus sequence, explaining induction of *PML* transcription following IFN stimulation (Stadler et al., 1995). Notably, deletion of ISRE from *PML* promoter is more derogatory for both type I and II IFN-dependent *PML* induction than GAS deletion which only modestly decreased *PML* sensitivity to type II IFN (Stadler et al., 1995). Moreover, downstream of type II IFN, IFN regulatory factor 8 (IRF8) binds to the ISRE within *PML* promoter leading to transcriptional upregulation of *PML* (Dror et al., 2007). IFN regulatory factor 3 (IRF3), on the other hand, has been shown to promote *PML* induction in an ISRE- and GAS-dependent way without the requirement of IFN itself (Kim et al., 2007). The *SP100* gene promoter also harbors ISRE and GAS consensus elements and is therefore IFN (both type I and type II)-inducible (Guldner et al., 1992; Grötzinger et al., 1996). Interestingly, IFN treatment also triggers enhanced SUMOylation of PML which coincides with an increase in PML NB size and number (Chelbi-Alix et al., 1995; Maroui et al., 2018). IFN-dependent PML SUMOylation also promotes Daxx recruitment to NBs (Kawai et al., 2003). IFN stimulation also causes a global increase in cellular SUMOylation. Mechanistically, PML, especially the PML III and PML IV isoforms, are important for this process by promoting Ubc9 SUMOylation and Ubc9 recruitment to PML NBs, further amplifying SUMO conjugation (Maroui et al., 2018). Another mechanism behind this global effect involves the induction of an ISG product Lin28B, which represses the expression of microRNAs (miRNAs) belonging to the let-7 family leading to the induction of let-7 targets such as the *sumo* transcripts (Sahin et al., 2014b).

Interestingly, PML is not only a downstream target of the IFN pathway, but has also emerged as a positive regulator of IFN signaling. PML promotes both IFN synthesis and IFN-induced expression of ISGs. PML II has specifically been reported to buttress type I IFN signaling through association with the transcription factor complexes containing IRF3, NFκB, and STAT1, thereby facilitating their DNA binding and subsequent expression of IFNβ and ISGs (Chen et al., 2015). PML also promotes STAT1/STAT2 activation in IFNβ treated cells and is possibly involved in recruiting STAT1/STAT2 containing interferon stimulated gene factor 3 (ISGF3) complex (containing phospho-STAT1, phospho-STAT2, and IRF9) to ISG promoters along with histone deacetylases (HDAC1, HDAC2) to favor transactivation (Kim and Ahn, 2015). Similarly, PML isoforms I–VI promote IFNγ-induced STAT1 phosphorylation leading to higher DNA binding of STAT1 and increased production of ISGs. In line with these findings, PML^–^/^–^ mouse embryonic fibroblasts (MEFs) were less efficient in IFNγ-mediated STAT1 DNA-binding activity compared to wild-type MEFs (El Bougrini et al., 2011). PML II also stabilizes the transcription factor CIITA (class II transactivator) which regulates IFNγ–induced major histocompatibility complex II (MHC II) gene transcription by protecting it from proteasomal degradation (Ulbricht et al., 2012). Interestingly, the MHC II gene locus is also spatially reoriented adjacent to PML NBs upon IFNγ stimulation, thereby facilitating a transcriptionally favorable environment for the MHC II expression (Gialitakis et al., 2010). Notably, PML-mediated IFN signaling involves its SUMOylation and the RING finger domain (El Bougrini et al., 2011). Additionally, many regulators of IFN synthesis and IFN-dependent signal amplification have now been reported to be modified by SUMO conjugation (El Motiam et al., 2020). Of importance, SUMO1 and SUMO3 exert distinct effects on the positive feedback loop between IFN signaling and PML, possibly by differentially promoting STAT1 SUMOylation and PML polySUMOylation (Maarifi et al., 2015). In addition to IFNs, production of other pro-inflammatory cytokines such as interleukin 1β (IL1-β) and interleukin 6 (IL-6) are positively regulated by PML as PML deficient cells are markedly inefficient in mounting this acute phase cytokine response (Lunardi et al., 2011; Lo et al., 2013). Consistently, PML knockout mice are insensitive to lipopolysaccharide (LPS)-induced acute shock response and also exhibit a dysregulated immune reaction to bacterial infection (Lunardi et al., 2011).

## PML NBs and Pathogens: A Dynamic Intricacy

Many DNA and RNA viruses exhibit cross-regulations with PML NBs. The spectrum of such interplays is wide with certain PML NB constituents curtailing viral gene expression while others are usurped for efficient viral propagation. Viruses have also evolved multifaceted measures to evade PML NB dependent intrinsic immune responses, thereby triggering viral gene expression required for their perpetuation. Moreover, PML NB integrity can also dictate the disease severity upon infection with pathogenic bacteria. In the following sections, we will describe the dynamic intricacies of viral and bacterial pathogens in modulating PML NBs. A profile of this intricate pathogen-host interaction is summarized briefly in Table 1.

**TABLE 1:** SUMO/SIM-dependent/independent interaction profiles of different pathogens with PML NB components.

Pathogen Family	Pathogens	Pathogenic Protein (Antagonist/Usurper)	Target with PML NBs (Subverted/Usurped)	Nature of interaction	Dependence on SUMO/SIM reported	Integrity of canonical PML NBs
*Herpesviridae*	HSV-1	ICP0	PML, Sp100	ICP0 acts as a viral StUbL and triggers proteasomal degradation of PML and Sp100	Yes	Affected
			ATRX	ICP0 triggers proteasome-dependent degradation of ATRX	No	Affected
	VZV	ORF61	PML	Disruption of PML NBs in ORF61 SIM-dependent manner without the reduction of PML protein stability	Yes	Affected
			Sp100	Degradation of Sp100 by ORF61 in RING finger dependent manner	No	Affected
		ORF23	PML IV	PML IV interacts with and sequesters VZV nucleocapsid protein ORF23	No	−
	HCMV	IE1	PML, Sp100	IE1 triggers loss of PML and Sp100 SUMOylation	Yes	Affected
		LUNA	PML	LUNA promotes SUMO deconjugation of PML	Yes	Affected
		pp71	Daxx-ATRX	pp71 interacts with and triggers proteasomal degradation of Daxx and also displaces ATRX from PML NBs	No	Affected
	HHV-6B	IE1	PML	IE1 co-localizes with all the nuclear PML isoforms; SUMO/SIM-dependent hyperSUMOylation of IE1 at PML NBs	Yes	Fostered
	KSHV	LANA1	Sp100	LANA1 promotes SUMOylation of Sp100 leading to its aggregation into PML NBs	Yes	Fostered
		LANA2	PML	LANA2 promotes PML SUMOylation and induces ubiquitin-proteasome dependent demise of SUMO-enriched PML	Yes	Affected
		K-Rta	PML	K-Rta acts as a viral StUbL and triggers proteasomal degradation of PML	Yes	Affected
		ORF75	Daxx-ATRX	ORF75 targets ATRX for proteasome-independent degradation and triggers redistribution of Daxx	No	Affected
	EBV	Zta	PML	Zta inhibits SUMO conjugation to PML by competing for the limiting pool of endogenous SUMO	Yes	Affected
		EBNA1	PML	EBNA1 induces degradation of PML through the ubiquitin-proteasome system	No	Affected
		BNRF1	Daxx-ATRX	BNRF1 binds to Daxx and outcompetes binding of Daxx to ATRX, thereby disrupting Daxx-ATRX complex formation	No	Affected
*Adenoviridae*	HAdV	E4orf3	PML II	E4orf3 interacts with PML II and reorganizes PML NBs into elongated nuclear tracks	No	Affected
		E2A	PML, Sp100	E2A undergoes SUMO conjugation, thereby fostering interaction with PML and Sp100A and convergence of PML NB tracks with the adenoviral RCs	Yes	−
		E1A	Ubc9, PML	E1A interacts with and affects polySUMOylation activity of Ubc9, disrupting SUMOylation of PML	Yes	Affected
		E1A-13S	PML II	E1A-13S interacts with PML II and trans-activates from adenoviral early promoter in a PML SIM-independent manner	No	−
		E1B-55K	Daxx	SUMOylated E1B-55K triggers proteasomal degradation of Daxx	Yes	Affected
		E1B-55K and E4orf6	ATRX	E1B-55K and E4orf6 co-recruits host Cullin complex and induces ubiquitin-proteasome dependent degradation of ATRX	No	Affected
		E1B-55K	p53	E1B-55K promotes SUMO1-dependent transient p53 recruitment to PML NBs; p53 is then expelled out of nucleus into cytoplasmic aggresomes	Yes	−
		E1B-55K	Sp100	E1B-55K triggers SUMOylation of Sp100A and sequesters it within PML containing nuclear tracks	Yes	−
		E1B-55K and E4orf6	p53	E1B-55K and E4orf6 E3 ubiquitin ligase complex triggers proteasomal degradation of p53	No	Affected
		VI	Daxx	VI interacts with and displaces Daxx from PML NBs to cytoplasm	No	Affected
	CELO	Gam1	SAE1/SAE2, PML	Gam1 induces ubiquitin-proteasome-dependent demise of the SAE1/SAE2 complex resulting in loss of SUMO conjugated PML	Yes	Affected
*Papillomaviridae*	HPV	L2	PML	L2 protein fosters association of the HPV genome with PML NBs in SIM-dependent manner	Yes	Fostered
*Picornaviridae*	EMCV	3C Protease	PML III	3C Protease triggers PML degradation in a proteasome- and SUMO-dependent manner	Yes	Affected
		3D Polymerase	PML IV	PML IV sequesters 3D Polymerase to PML NBs, thereby impairing viral perpetuation	No	−
	Poliovirus		p53	Poliovirus induces PML-dependent p53 activation which results in mobilization of apoptotic cascade	Yes	Fostered
*Rhabdoviridae*	Rabies virus	P	PML	P delocalizes PML III from nuclear to cytoplasmic puncta where both proteins co-localize	No	Affected
*Arenaviridae*	LCMV	Z	PML	Z protein induces redistribution of PML from nuclear condensates to cytoplasmic aggregates	No	Affected
*Flaviviridae*	DENV	NS5	PML	NS5 co-localizes with PML III and IV at PML NBs and promotes accelerated degradation of these PML isoform	No	Affected
	HCV	Core protein	p53	HCV core protein gets recruited to PML NBs by interacting with PML IV and inhibits the co-activator activity of PML IV on p53	No	−
*Retroviridae*	HIV-1		PML, Daxx	PML NBs rapidly relocalize from nuclear to cytoplasmic aggregates	Yes	Affected
		Vpu	PML	Vpu interacts with RanBP2 to inhibit the RanBP2–RanGAP1*SUMO1–Ubc9 SUMO E3 ligase, thereby affecting PML NB integrity	Yes	Affected
*Listeriaceae*	*Listeria monocytogenes*	LLO	Ubc9, PML, Sp100	LLO promotes proteasome-independent degradation of Ubc9 and loss of SUMO conjugated PML and Sp100	Yes	Affected
*Streptococcaceae*	*Streptococcus pneumoniae*	PLY	Ubc9, PML	PLY promotes degradation of Ubc9 and loss of SUMO conjugated PML	Yes	Affected
*Clostridiaceae*	*Clostridium perfringens*	PFO	Ubc9, PML	PFO promotes degradation of Ubc9 and loss of SUMO conjugated PML	Yes	Affected
*Enterobacteriaceae*	*Shigella*	T3SS	Ubc9, PML	T3SS triggers proteasome-dependent Ubc9 destabilization; the number of PML NBs increases during infection	Yes	Altered

### DNA Viruses

#### Herpesviruses

##### Herpes Simplex Virus-1 (HSV-1)

Small ubiquitin-related modifier-dependent dynamics of PML NBs shapes the outcome of infection with HSV-1, an alphaherpesvirus of the *Herpesviridae* family which generally causes oral sores. Nuclear entry of the viral genomic DNA triggers host cells to mount an intrinsic antiviral response. An essential facet of this response is the silencing of the viral gene expression and onset of latency. This latent reservoir of virus signifies an evolutionary achievement of the virus as it bypasses immune clearance and has the potential to get reactivated periodically leading to disease manifestation and viral transmission. Interestingly, SUMO-dependent regulations are important for initiating this latency. The incoming viral genome, which is a linear molecule of DNA, adsorbs a plethora of both pro- and antiviral host cellular factors (Dembowski and DeLuca, 2018). These include the H3.3 histone chaperone complex Daxx/ATRX which further enables subsequent entrapment of the viral DNA (vDNA) within PML NBs. Other PML NB resident proteins, such as PML and Sp100, as well as the nuclear DNA sensing protein IFI16 also deposit onto the viral genome, where they co-localize with ICP4, an immediate early gene that serves as a marker for incoming HSV-1 genomes (Everett, 2015; Diner et al., 2016; Alandijany et al., 2018; Cabral et al., 2018). Interestingly, SUMO modification of PML and SIMs of PML, Sp100 and Daxx are pivotal for the association of vDNA with PML NBs (Cuchet-Lourenço et al., 2011). Engulfment of the vDNA within PML NBs coincides with the repressive epigenetic modification of the histones (H3K9me3, H3K27me3) on vDNA leading to a transcriptionally inactive chromatin environment (Lee et al., 2016; Cabral et al., 2018; Cohen et al., 2018). The importance of SUMO conjugation to PML and PML-associated NB components is further supported by the finding that host proteins, which promote SUMO conjugation to PML and PML-NB associated proteins curtail HSV-1 infection (Boutell et al., 2011; Brown et al., 2016; Conn et al., 2016). For example, PIAS1, which localizes to PML NBs and acts as a SUMO E3 ligase on PML, contributes to HSV-1 restriction (Brown et al., 2016; Li et al., 2020).

During infection with the wild type virus, the PML NB association of vDNA is transient as the HSV-1 protein ICP0 counteracts this quiescence by dispersal of PML NBs. ICP0 is dispensable for the establishment and maintenance of HSV-1 latency but very critical for the productive rejuvenation of the viral genome from latency and further onset of lytic replication (Leib et al., 1989; Cai et al., 1993; Halford and Schaffer, 2001). Infection with an ICP0 null mutant results in a more persistent association between HSV-1 genome and PML NBs, whereas expression of ICP0 triggers dismantling of PML NB associated proteins from vDNA (Everett et al., 2013a; Alandijany et al., 2018; Cabral et al., 2018). This results in a reduction of histone H3 deposition and enhanced acetylation of remaining histone H3 on vDNA to promote viral transcription (Cliffe and Knipe, 2008; Ferenczy et al., 2011; Lee et al., 2016; Cabral et al., 2018). These observations support a model, in which sequestration of the HSV-1 genome in PML NBs induces repression of viral gene expression, while ICP0-mediated destruction of NBs counters this process.

**FIGURE 3:**
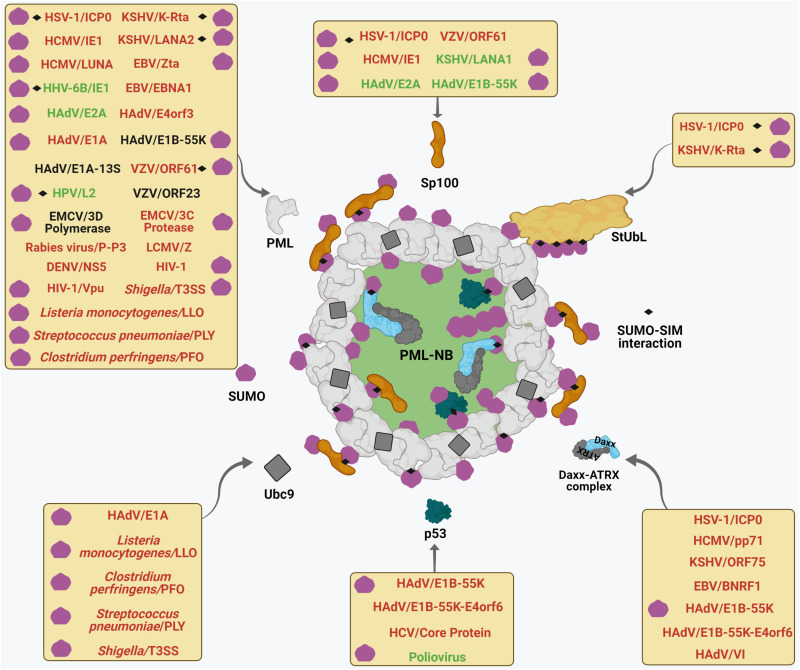
Multifaceted interactions of viruses and bacteria with PML NBs. Different viruses and bacteria target mature PML NBs. Each box represents viral and bacterial components which target specific PML NB components (shown by curved arrows) to either foster (text-colored Green) or perturb (text-colored Red) PML NB integrity or are neutral (text colored Black) on PML NB. The pathogen-PML NB interactions which are SUMO-regulated and/or SUMO-SIM affinity driven are designated by using corresponding symbols within each box. For further details, please refer to the main body of the article.

**FIGURE 4:**
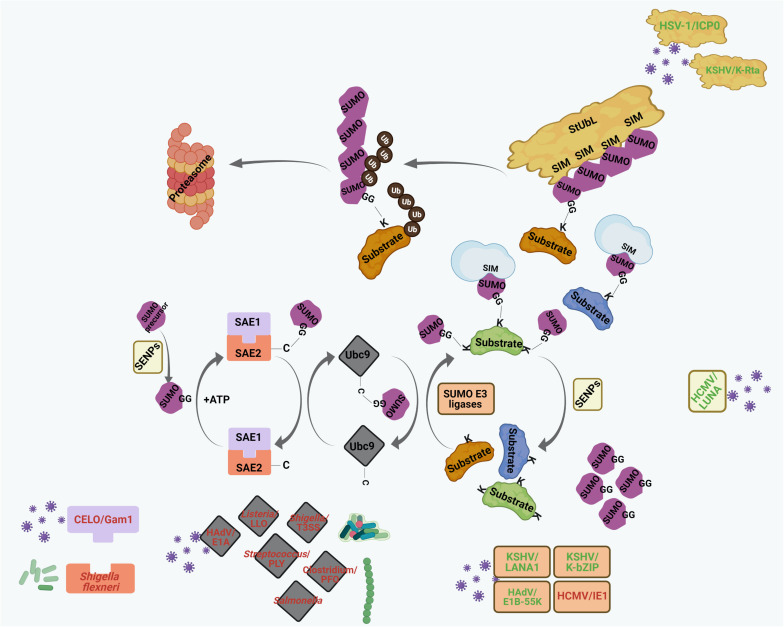
Interplays between different pathogenic components and host cellular SUMO machinery regulating PML NB integrity. Many viral and bacterial factors manipulate with the host cellular SUMO conjugation/deconjugation system to modulate PML NBs. The process of SUMO conjugation and deconjugation as in Figure 2 is shown here. Components of different pathogens are represented within specific colored shapes which are look-alike with their targets within the SUMO system (such as E1, E2, E3, SENPs, and StUbLs). The components which mimic targets within the SUMO machinery are text-colored green whereas those which destabilize/antagonize targets within the SUMO system are text-colored red. For further details, please refer to the main body of the article.

Core to the functions of ICP0 is its ability to act as a viral StUbL, thereby triggering RING finger-dependent proteolytic ubiquitylation of SUMO-conjugated proteins (Figures 3, 4), including PML (Boutell et al., 2003, 2011; Lanfranca et al., 2014). Accordingly, mutants of HSV-1 which lack ICP0 or express a ligase deficient ICP0, have a dramatically lower probability of initiating a productive infection in restrictive cell types (Everett et al., 2004). Reminiscent to the StUbLs RNF4 or RNF111, ICP0 harbors a RING-domain and 7 putative SIM-Like Sequences (SLS). Accordingly, IPC0 possesses biochemical properties which are similar to host cellular StUbLs (Everett et al., 1998; Boutell et al., 2003, 2011; Sloan et al., 2015). The structure of ICP0 SLS4 (residue 362–367) with SUMO has been resolved by NMR, revealing a cooperation between ICP0 phosphorylation domains (FHA [67-pTELF-70] and Phos2) for recognition of the SUMOylated proteins (Boutell et al., 2008; Mostafa et al., 2013; Hembram et al., 2020). Therefore, both the ICP0 RING-finger and phosphorylation are important for the degradation of SUMOylated proteins and successful reactivation of HSV-1 from latency (Mostafa et al., 2011, 2013; Vanni et al., 2012). One specific SIM in the central region of ICP0 is absolutely essential for degradation of an array of SUMOylated host proteins, including PML and Sp100 (Figure 3; Everett et al., 1998, 2009, 2014; Müller and Dejean, 1999).

ICP0 also induces the degradation of ATRX and IFI16 (Figure 3; Jurak et al., 2012; Orzalli et al., 2012, 2016; Diner et al., 2015b), but turnover of ATRX and IFI16 occurs with delayed kinetics relative to that of PML (Jurak et al., 2012; Cuchet-Lourenço et al., 2013), possibly indicating that degradation of host components occurs sequentially as infection progresses (Merkl and Knipe, 2019). This is consistent with the step-wise accumulation of cellular factors on vDNA throughout the initiating cycle of HSV- 1 infection (Dembowski and DeLuca, 2015, Dembowski and Deluca, 2017). Recent studies have identified the histone H3.3 chaperone protein HIRA to restrict HSV-1 infection following the onset of vDNA replication, a host response antagonized by ICP0 through the nuclear dispersal of HIRA (McFarlane et al., 2019). Thus, multiple histone H3.3 chaperone proteins (Daxx/ATRX and HIRA) can restrict the replication of ICP0 null HSV-1 at independent phases of infection (Rodríguez et al., 2020). Notably, ICP0 null mutants exhibit reduced plaque forming efficiency which can be reversed by co-depletion of either PML, Sp100, Daxx or ATRX (Everett et al., 2006, 2008; Lukashchuk and Everett, 2010). Absence of a single component does not affect the recruitment of other factors onto HSV-1 genomes, indicating their independent deposition onto the viral genome (Everett et al., 2006, 2008; Lukashchuk and Everett, 2010). Consistent with this, simultaneous depletion of PML and Sp100 enhances ICP0-null HSV-1 propagation greater than single depletion of each factor (Everett et al., 2008). However, another study reported knockdown of PML to enhance wild type HSV-1 propagation (Diner et al., 2015a). Interestingly, SIM-deficient mutants of PML and Daxx are not recruited to vDNA and are incapable of reproducing the repression of ICP0 null mutant HSV-1 viruses (Cuchet-Lourenço et al., 2011), indicating that SIM-dependent PML NB recruitment to HSV-1 DNA during the initial phase of infection is needed to create a transcriptionally repressive environment. Of note, though Sp100 also contributes to the repression of gene expression from null ICP0 HSV-1 virus (Everett et al., 2008, 2009; Glass and Everett, 2013), direct implications of Sp100 on the epigenetic regulation of viral chromatin or modulating innate immune signaling are yet to be determined.

Apart from PML NB resident proteins, a number of host cellular SUMO-modified proteins have now been identified to be targeted by ICP0 (Everett et al., 1998; Chelbi-Alix and de Thé, 1999; Boutell et al., 2011; Sloan et al., 2015). For many of these targets, however, specificity and relevance to HSV-1 replication have yet to be determined, implicating a scenario where at least some of these proteins may be the subjects of “bystander” degradation. Importantly, upon degradation of MORC3 which is a SUMO-conjugated PML NB client targeted by ICP0, recruitment of other PML NB components PML, Sp100, Daxx on to vDNA is also inhibited, substantiating the importance of these foci in defense against invading pathogens (Sloan et al., 2016).

In addition to disarming PML NB-oriented intrinsic immunity, ICP0 also inactivates innate immune defenses early in the infectious cycle through the degradation of cellular PRRs. Detection of viral nucleic acid by host PRRs can elicit a signal transduction cascade leading to generation of IFNs and mobilization of IFN-dependent downstream signaling to produce hundreds of ISGs with antiviral potency. Interestingly, null ICP0 HSV-1 mutants are hypersensitive to IFN (Leib et al., 1999; Mossman et al., 2000; Härle et al., 2002; Everett et al., 2004), indicating that ICP0 limits the IFN-dependent innate immune response. PML II and IV directly influence the induction of cytokines and ISGs by facilitating the loading of transcription factors (including IRF3, NF-κB, and STAT1) onto cellular gene promoters, possibly explaining the IFN hypersensitivity phenotype of null ICP0 HSV-1 mutants (Chee et al., 2003; El Asmi et al., 2014; Chen et al., 2015; McFarlane et al., 2019). Subsequent studies revealed that ICP0 disarms nuclear PRR by degrading IFI16 which recognizes viral dsDNA and signals via STING-dependent pathway and also by triggering decay of DNA-PKcs (Orzalli et al., 2012, 2015, 2016; Cuchet-Lourenço et al., 2013; Diner et al., 2016; Burleigh et al., 2020). Interestingly, a recent study showed a distinct temporal segregation between the entrapment of HSV-1 DNA by PML NBs upon its nuclear entry and the subsequent deposition of IFI16 leading to ISG expression (Alandijany et al., 2018). While the former contributes to the host cellular intrinsic defense measure by triggering viral latency, the latter is related to the host innate immune response. Moreover, PML NB entrapment of vDNA upon its nuclear entry was shown to be independent of IFI16 deposition onto the viral genome which was shown to require vDNA replication. PRR sensing by IFI16 during null ICP0 HSV-1 infection correlates with IFI16 forming nuclear filaments on vDNA in association with PML following the saturation of PML NBs under high genome loads (Cuchet-Lourenço et al., 2013; Alandijany et al., 2018; Merkl and Knipe, 2019). A close functional relationship between innate and intrinsic immunity is further evidenced when resident PML NB proteins (PML, Daxx and ATRX) have been shown to act cooperatively with IFI16 to restrict null ICP0 HSV-1 gene expression that correlates with repressive histone signatures (H3K9me3 and H3K27me3) on viral chromatin (Everett et al., 2008; Lee et al., 2016; Cabral et al., 2018; Merkl et al., 2018).

##### Varicella-Zoster Virus (VZV)

Another alphaherpesvirus VZV is the etiological agent of chickenpox and the reactivation disease known as shingles (Gilden et al., 2015). Disruption of PML NBs without the reduction of PML protein stability itself has been evidenced in VZV infected cells both in cell culture based studies and *in vivo* (Kyratsous and Silverstein, 2009; Reichelt et al., 2011). The viral trigger has been found to be the protein ORF61 which is homologous to HSV-1 ICP0 in having a RING finger and SIMs (Figure 3; Moriuchi et al., 1992; Everett et al., 2010; Wang et al., 2011). Unlike ICP0, however, StUbL activity of ORF61 has not yet been formally determined. Nonetheless, Sp100 is targeted for degradation by ORF61 especially during early hours of infection in a ORF61 RING finger-dependent manner (Figure 3; Kyratsous et al., 2009; Kyratsous and Silverstein, 2009; Walters et al., 2010). The significance of this process on VZV life cycle has not been addressed yet. Interestingly, three SIMs within ORF61 were found to be indispensable for targeting of PML NBs. In response to the ORF61 SIM mutant virus, viral replication in skin was severely hampered, the typical VZV lesions were reduced, and viral spread in the epidermis was limited (Wang et al., 2011), suggesting that the SUMO binding function of ORF61 is critical for overcoming the anti-viral effects of the PML bodies. Of interest, as a part of the dynamic host-virus interactions, the persisting PML NBs in VZV infected cells can also mount a potent anitiviral response by sequestering viral nucleocapsids. Mechanistic studies revealed that PML IV interacts with the VZV capsid protein ORF23 to enable this sequestration and to prevent nuclear egress of viral nucleocapsids, thereby inhibiting formation of infectious virions (Figure 3; Reichelt et al., 2011). However, a direct involvement of host cellular SUMO system in this antiviral defense mechanism has remained elusive.

##### Human Cytomegalovirus (HCMV)

Human cytomegalovirus is a betaherpesvirus which also has the potency to go into latency or to resume lytic reactivation and proliferation. Importantly, transcription of the viral immediate early (IE) genes is crucial for switching the viral infection cycle toward lytic proliferation. Major HCMV IE genes are transcribed under the control of the major immediate early promoter (MIEP), which is only transactivated by a viral protein residing within the tegument layer of infectious virions.

Dynamic SUMO modifications have been reported to contribute to the regulations of intrinsic and innate immune defenses against infection with HCMV. The initial observation that herpesviral DNA associates with PML NBs upon its nuclear entry led to the hypothesis that these condensates are usurped by cytomegalovirus and other herpesviruses for productive initiation of the viral IE gene expression (Maul et al., 1996; Ishov and Maul, 1996; Ishov et al., 1997). Subsequent investigations revealed that these spatial proximity and entrapment of vDNA within PML NBs during initial hours of infection constitute a major arm of the cellular intrinsic antiviral defense (Saffert and Kalejta, 2006; Tavalai et al., 2006, 2008; Woodhall et al., 2006; Adler et al., 2011). This is enabled by creating a transcriptionally–incompetent chromatin environment on vDNA leading to silencing of viral IE gene expression. Agreeably, knocking down expression of individual PML NB components such as PML, Daxx, and Sp100 in primary human fibroblasts showed a de-repressing effect on HCMV IE gene expression at low multiplicity of infection (Saffert and Kalejta, 2006; Tavalai et al., 2006, 2008; Woodhall et al., 2006; Adler et al., 2011). Moreover, simultaneous depletion of more than one PML NB component has additive effects on the number of IE-positive cells than when single knock down was achieved, suggesting that multiple NB components independently contribute to restriction of HCMV lytic replication (Tavalai et al., 2008; Adler et al., 2011). Interestingly, PML NB components play a crucial role in epigenetic modulations of the MIEP, leading to repression of the viral IE gene expression (Groves et al., 2009; Sinclair, 2010). The contribution of Daxx to trigger epigenetic quiescence of viral gene expression is evident from analyzing the chromatin signatures around the MIEP in Daxx-deficient cells (Woodhall et al., 2006). Daxx has also been shown to recruit HDACs and ATRX to the viral promoter to execute transcriptional repression (Saffert and Kalejta, 2006; Lukashchuk et al., 2008). Not surprisingly, therefore, inhibition of HDACs by trichostatin A or depletion of ATRX had a de-repressing effect on HCMV IE gene expression (Saffert and Kalejta, 2006; Lukashchuk et al., 2008). In addition to Daxx, PML has also been reported to interact with HDACs and histone methyl transferases (HMTs) and Sp100 with heterochromatin protein 1 (HP1) (Seeler et al., 1998; Wu et al., 2001; Di Croce et al., 2002; Wilcox et al., 2005; Carbone et al., 2006). However, implications of such interactions in mediating epigenetic silencing of viral genome are yet to be addressed experimentally. Although some mechanistic details are still unclear, the ensemble of these data indicate that - in analogy to HSV1- the sequestration of the HCMV genome in PML NBs mediates the repression of viral gene expression through epigenetic silencing of the genome.

Importantly, however, HCMV has also developed countermeasures to rejuvenate its life cycle and to maintain perpetuation. There are three effector proteins, the tegument protein pp71, the Latency Unique Natural Antigen (LUNA) protein and the major immediate-early protein IE1, which can affect the integrity of PML NBs (Figure 3). The mechanisms employed, however, are different. After being transported to the nucleus during infection of terminally differentiated cells such as fibroblasts and epithelial cells, pp71 interacts with and triggers proteasomal degradation of Daxx and also displaces ATRX from the PML NB condensates (Figure 3; Hofmann et al., 2002; Saffert and Kalejta, 2006; Lukashchuk et al., 2008). This further relieves the Daxx-mediated transcriptional repression on the viral genome leading to expression of viral IE1 from MIEP and productive viral infection. A pp71-null HCMV virus has a major defect in IE gene expression and productive replication (Bresnahan and Shenk, 2000; Cantrell and Bresnahan, 2006). Following infection of incompletely differentiated myeloid cells such as CD34^+^ hematopoietic progenitor cells (HPCs) and monocytes, pp71 remains trapped in cytoplasmic endosomes (Penkert and Kalejta, 2010, 2013; Lee et al., 2019; Lee and Kalejta, 2019; Kalejta and Albright, 2020). As a result, Daxx is not degraded, thereby facilitating histone deposition and assembly of repressive heterochromatin on viral genomes in conjunction with ATRX and the KAP1/SetDB1/HDAC co-repressor complex. Heterochromatin assembly results in repression of the MIEP allowing for the establishment of latency (Murphy et al., 2002; Groves et al., 2009; Sourvinos et al., 2014; Lee et al., 2015; Rauwel et al., 2015; Albright and Kalejta, 2016; Kalejta and Albright, 2020). Importantly, pp71 mutants which cannot interact with Daxx are also unable to activate an MIEP reporter (Hofmann et al., 2002), substantiating the importance of pp71-Daxx interaction for Daxx repression and transactivation from MIEP. Apart from Daxx degradation, pp71 also triggers Daxx SUMOylation (Hwang and Kalejta, 2009). SUMOylated Daxx is unable to interact with NFκB and therefore fails to prevent NFκB acetylation and activation (Croxton et al., 2006; Park et al., 2007; Kim et al., 2017). This leads to NFκB-dependent enhanced MIEP activity (DeMeritt et al., 2004; Liu et al., 2010; Yuan et al., 2015) and efficient IE gene expression at least in certain contexts.

Although the pro-viral relevance of pp71 in initiating viral IE gene expression to induce a *de novo* lytic infection is well studied, importance of pp71 in mediating viral rejuvenation from latency and its role of NB remodeling in this process remains questionable (Kalejta and Albright, 2020). Interestingly, a recent report has highlighted disruption of PML NBs in a pp71-independent manner by the viral LUNA protein (Poole et al., 2018). LUNA is encoded from a transcript antisense to the viral *UL81* and *UL82* genes (which also codes for pp71) (Bego et al., 2005) and is expressed not exclusively during latency but also during lytic infection (Keyes et al., 2012). LUNA has been shown to promote HCMV reactivation (Keyes et al., 2012) by triggering dismantling of PML NBs through a proposed deSUMOylase activity (Figures 3, 4; Poole et al., 2018). Mechanistically, LUNA was proposed to act as a cysteine protease, thereby catalyzing SUMO deconjugation from PML and possibly explaining the loss of PML NB integrity in presence of this HCMV protein (Figure 3). Substituting a potential cysteine residue in the predicted catalytic center completely abrogated isopeptidase activity as well as PML NB disruption by LUNA and this phenomenon coincides with impaired viral reactivation from latency. Of interest, reactivation deficiency of LUNA-null or LUNA catalytically dead virus mutants is rescued in PML-deficient cells, further substantiating LUNA-dependent deSUMOylase activity on PML to be important for HCMV rejuvenation from latency (Poole et al., 2018).

During a productive lytic infection, tegument-delivered pp71 initiates and sustains MIEP transcription long enough for the *de novo* synthesized IE1 protein to promote prolonged lytic phase gene expression (Vardi et al., 2018) and the completion of productive replication. IE1 protein employs a SUMO-dependent mechanism to disrupt PML NBs (Figure 3). IE1 accumulates at PML NB foci and depletes specifically the SUMO-modified isoforms of PML and Sp100 in a proteasome-independent manner without perturbing global SUMOylation (Figure 3; Müller and Dejean, 1999; Xu et al., 2001; Lee et al., 2004; Tavalai et al., 2011; Everett et al., 2013a; Scherer et al., 2013). IE1 itself is SUMOylated on K450 (Xu et al., 2001; Lee et al., 2004; Nevels et al., 2004; Sadanari et al., 2005), but the functional significance of this modification is poorly understood. Expression of an IE1 K450R mutant shows decreased transcripts and protein expression of IE2, the primary viral transactivating protein for HCMV early and late gene expression, suggesting SUMO modification of IE1 to possibly impact IE2 expression (Nevels et al., 2004). Interestingly, SUMOylation of IE1 is dispensable for its PML NB disrupting function (Xu et al., 2001; Spengler et al., 2002; Lee et al., 2004). However, this disruption requires a direct physical interaction between an extended interaction interface of IE1 globular core domain and the coiled-coil domain of PML (Ahn et al., 1998; Lee et al., 2004; Scherer et al., 2014). How IE1 triggers loss SUMO-conjugated isoforms of PML and Sp100 has recently been elucidated in both cell-based assays and *in vitro* SUMOylation assay. IE1 was found to sensitize only the *de novo* SUMOylation at K160 of PML after binding to PML but not the deSUMOylation of pre-SUMO-modified PML (Schilling et al., 2017). Moreover, IE1 also prevented As_2_O_3_-mediated hyperSUMOylation of PML at K160, thereby blocking PML degradation. Since IE1 did not interfere with the coiled-coil-mediated PML dimerization, it has been speculated that IE1 impairs PML autoSUMOylation either by directly abrogating the E3 ligase function of PML (Figure 4) or by preventing access to SUMO sites (Schilling et al., 2017). Besides PML and Sp100, IE1 also targets Daxx to antagonize Daxx-mediated transcriptional repression of the viral genome (Reeves et al., 2010). However, the mechanistic details of IE1-Daxx interaction has not been addressed so far. Moreover, IE1 also induces degradation of SUMO unmodified Sp100 in a proteasome-sensitive way in late stages of infection. This event was revealed to be independent of PML NB disruption (Kim et al., 2011).

Interestingly, IE1-mediated PML NB disruption not only has antiviral significance from the perspective of disarming host cellular intrinsic defense measures but also has implications in abrogating innate immune response (Kim and Ahn, 2015; Scherer et al., 2015). SUMOylation status of IE1 regulates IE1-dependent inhibition of innate immune signaling. Unmodified IE1 interacts with STAT1 and STAT2 in the nucleus and decreases binding of these transcription factors to target promoters, leading to repression of ISG transcription (Paulus et al., 2006; Huh et al., 2008; Krauss et al., 2009). Therefore, in comparison to wild-type HCMV, IE1-deleted HCMV shows hypersensitivity to IFN. Interestingly, SUMOylated IE1 has reduced affinity for STAT2 and is less efficient in inhibiting STAT2-dependent ISG transcription (Huh et al., 2008). In addition to STATs, IE1 also antagonizes type I IFN signaling by interacting with PML, thereby sequestering ISGF3 into a functionally inactive complex incapable of DNA binding and ISG transactivation (Kim and Ahn, 2015).

Structural characterization of the IE1 core (Scherer et al., 2014) has revealed it to be a compact, contiguous domain that is highly sensitive to small deletions and point mutations. A single leucine-to-proline exchange (L174P) within the core domain of IE1 at residue 174 hampers the structural integrity and stability of the protein. Consequently, the L174P mutant is incapable of triggering PML NB disruption and has a growth defect that phenocopies IE1-deleted virus (Scherer et al., 2015, 2017). Only very recently, a novel IE1 mutant (IE1cc172–176) has been characterized, which is selectively deficient in interacting with PML, inhibiting PML SUMOylation, and therefore incapable of PML NB localization and disruption without losing overall structural stability (Paulus et al., 2020). Very interestingly, this mutant has been shown to support viral replication almost as efficiently as its wild-type counterpart. Subsequent functional analysis of IE1cc172–176 revealed that this protein is hypermodified by mixed SUMO chains and that IE1 SUMOylation depends on nucleosome rather than PML binding. IE1 mutant which cannot bind to nucleosome (IE1dl476–491) and show reduced chromatin association was revealed to be poorly SUMO-modified, possibly suggesting that IE1 SUMOylation takes place on chromatin. The chromatin-associated E3 SUMO ligase PIAS1 was proposed to SUMOylate IE1 (Paulus et al., 2020). Indeed, PIAS1 has been reported to interact with IE1, to enhance IE1 SUMOylation (Kim et al., 2014), and to act as a SUMO E3 ligase for many transcription factors and chromatin associated proteins (Wu et al., 2014). It was speculated that PIAS1-directed SUMOylation might promote dissociation of the viral protein IE1 from chromatin surface (Paulus et al., 2020). This also explains why IE1 is SUMOylated at nucleosomes but localizes throughout the nucleus. Of interest, with the use of this selective IE1 mutant, not only HCMV IE1-induced deSUMOylation of Sp100 was uncoupled from that of PML, but also upregulation of IFN signaling and ISG expression were observed (Paulus et al., 2020). However, speculation such as disruption of PML NBs upon viral infection is linked to activation rather than inhibition of innate immunity is subject to further studies in other virus infection scenario.

##### Human Herpesvirus-6 (HHV-6)

Promyelocytic leukemia and PML NBs have also proven to be critical regulators of the betaherpesvirus human herpesvirus 6A and B (HHV-6A/B). HHV-6 infects peripheral blood mononuclear cells (PBMCs) and various T-cell lines where it can either establish latent infection or can replicate as a part of its lytic programme. Notably, HHV6 integrates its genome into the telomeric regions of host chromosomes for prolonged maintenance. Importantly, PML NBs and the SUMO machinery were shown to be involved in controlling viral latency as well as viral integration. In case of HHV6A, it was demonstrated that knocking down components of PML NB such as PML, Daxx, and Sp100 significantly increased viral lytic replication and late gene expression. Consistently, progressive lytic infection was found to reduce the number of PML NBs within infected cells with concomitant increase in the puncta size without triggering complete disassembly of these speckled structures (Sanyal et al., 2018). These findings are in line with a role of PML NBs in restricting lytic replication of HHV-6A.

Work on HHV-6B has recently revealed a surprising facet of PML and the SUMO machinery in integrating the HHV-6B genome into the telomeric regions of host chromosomes for prolonged maintenance (Collin et al., 2020). Further, the HHV-6B trans-activator protein IE1 which undergoes SUMOylation at K802 and localizes to PML NBs has emerged as the viral regulator in this process (Figure 3; Gravel et al., 2002, 2004; Stanton et al., 2002; Collin et al., 2020). Mechanistically, IE1 co-localizes with all the nuclear PML isoforms to subsequently get hyperSUMOylated by SUMO1 (involving K802 and possibly other SUMO conjugation sites), but not SUMO2/3, in presence of PML and intact PML NBs. A putative SIM as well as the primary SUMO accepting site K802 proved to be essential for IE1 hyperSUMOylation and PML NB recruitment. Moreover, PML was also found to mediate IE1 localization to telomeres and PML depletion sensitized telomeric recruitment of IE1 as well as HHV-6B genomic integration into the host chromosomes (Collin et al., 2020).

##### Kaposi’s Sarcoma-associated Herpesvirus (KSHV)

Kaposi’s sarcoma-associated herpesvirus is a gammaherpesvirus which is capable of establishing latent infection and can get periodically activated to undergo lytic replication and production of progeny viruses. Much alike to the other herpesviruses, PML NB-mediated restriction of the infecting viral genome has been shown to be important for triggering quiescence of KSHV genome. Latency-associated nuclear antigen LANA1 is the primary viral product during latency and has been reported to bind to a series of cellular gene promoters to modulate gene transcription. LANA1 promotes SUMOylation of Sp100 leading to its aggregation into PML NBs (Figure 3; Günther et al., 2014), thereby fostering structural integrity of these structures. LANA1 has a SUMO2-specific SIM which aids in SUMOylation of LANA1 at K1140 residue and also enables recruitment of SUMO2-modified KAP1 together with the Sin3A-containing transcriptional repressor complex. LANA1-KAP1 association maintains viral latency by silencing the expression of viral lytic trans-activator K-Rta. Deletion of SIM from LANA1 abrogates this association, thereby triggering loss of viral episomal genome maintenance and loss of repression of K-Rta expression (Cai et al., 2013). A recent study also demonstrated that inhibition of the LANA SIM motif by a small molecule Cambogin exerted significant antagonistic effects on KSHV persistent infection and proliferation of KSHV-latently infected cells both *in vitro* and *in vivo* (Ding et al., 2019). Notably, LANA1 also exerts E3 SUMO ligase-like activity (Figure 4) by enhancing SUMOylation of histones H2A-H2B in a SIM-dependent manner through recruiting the SUMO-Ubc9 intermediate. This likely contributes to chromatin condensation, repression of viral genes and maintenance of viral latency (Campbell and Izumiya, 2012). LANA1 SUMOylation is controlled by the SUMO isopeptidase SENP6 and LANA1 and SENP6 expression are mutually interdependent (Lin et al., 2017). SUMOylated LANA1 further increases LANA1 expression whereas SENP6-mediated deSUMOylation reduces LANA1 expression. Interestingly, LANA1 itself binds to SENP6 promoter to reduce SENP6 expression. This leads to an increased pool of SUMO-modified LANA1 which further maintains its high expression and promotes viral latency. During *de novo* infection, overexpressing SENP6 has been shown to impair viral latency owing to diminished LANA protein levels which further led to enhanced viral gene expression (Lin et al., 2017).

Another KSHV protein K-bZIP that belongs to the basic region-leucine zipper family of transcription factors, usurps host cellular SUMOylation machinery to exert its function (Izumiya et al., 2005). K-bZIP acts as a transcriptional repressor of the viral genome and aids in maintaining viral latency. Interestingly, K-bZIP has been shown to interact with Ubc9 and to potentially serve as a viral SUMO E3 ligase (Figure 4). K-bZIP’s proposed SUMO E3 ligase activity was revealed to be specific for SUMO2/3 conjugation, possibly implicating a role in promoting polySUMOylation of targets. K-bZIP is itself modified at K158 both *in vitro* and *in vivo* (Chang et al., 2010); but a SUMO-deficient mutant of K-bZIP does not show altered protein stability or localization to PML NBs. Further, SUMOylation of K-bZIP is mostly dispensable for the trans-repression activity. However, trans-repression requires interaction of K-bZIP with the catalytically active Ubc9 (Izumiya et al., 2005). Though detailed molecular explanations are lacking, K-bZIP has been shown to induce SUMO2/3 conjugation of transcription factors at the viral promoters, resulting in repression of viral transcription and establishment of viral latency (Yang et al., 2015a). During KSHV reactivation, enrichment of SUMO2/3 association with the promoter regions of both the viral and cellular genomes has been reported (Chang et al., 2013; Yang et al., 2015a). Though awaiting experimental affirmation, K-bZIP may act as a SUMO conjugating agent at cellular promoters thereby repressing cellular innate immune response (such as expression of ISGs) (Chang et al., 2013). At viral genomes, K-bZIP fosters enhanced association of SUMO2/3 with the viral DNA, thereby providing another check for the virus to skew its life cycle back toward latency before lytic commitment. Infection with a virus harboring a SUMO E3 ligase mutant of K-bZIP does not show this effect and has increased proliferation capabilities. Interestingly, knocking down SUMO2/3 expression phenocopied this effect (Yang et al., 2015a). Of note, K-bZIP also harbors a SIM that binds exclusively to SUMO2/3 (Chang et al., 2010). Direct implications of K-bZIP in usurping PML NB components for SUMOylation to induce KSHV latency, however, are lacking so far.

Kaposi’s sarcoma-associated herpesvirus employs several strategies to target PML NB integrity for inducing viral reactivation and lytic rejuvenation. The first KSHV protein which can antagonize PML NBs during *de novo* infection is one of the viral tegument proteins ORF75 which specifically targets ATRX for degradation in a proteasome-independent manner and triggers redistribution of Daxx, without hampering other PML NB constituents (Figure 3; Full et al., 2014). PML NBs become lesser in number and appear as irregular-shaped larger structures in cells where KSHV-ORF75 is expressed alone. A mutant KSHV lacking the entire *ORF75* gene is replication-deficient in cell culture and cannot promote viral gene expression, though much of it can be attributable to the importance of this KSHV protein in structural integrity of the virus (Full et al., 2014).

A robust SUMO-dependent way that KSHV adopts to disrupt PML NB aggregates during the onset of lytic replication requires the viral protein K-Rta. This KSHV trans-activating protein exerts StUbL activity by targetting SUMOylated proteins for ubiquitylation and proteasomal degradation (Figures 3, 4; Izumiya et al., 2013). K-Rta possesses a RING finger domain and multiple SIMs with affinity toward SUMO2/3 multi-/polymers (Figure 4). SUMOylated PML is one of the primary targets of K-Rta, explaining the disruption of PML NBs by K-Rta (Figure 3). Accordingly, SIM-mutated K-Rta was shown to be incapable of triggering PML NB disruption (Izumiya et al., 2013). Another important target of K-Rta is K-bZIP. K-bZIP acts as a transcriptional repressor of K-Rta, thereby impairing viral transactivation and lytic rejuvenation. Therefore, degradation of K-bZIP by the StUbL activity of K-Rta counteracts the repressive action of K-bZIP. Indeed, SIM-mutated K-Rta showed reduced replication potential and attenuated potency of viral transactivation by K-Rta (Izumiya et al., 2013).

Another KSHV protein which disrupts PML NBs is LANA2 (vIRF-3). LANA2 promotes PML SUMOylation and induces proteolytic ubiquitylation followed by proteasomal demise of SUMO-enriched PML, again leading to dissolution of PML NBs (Figure 3; Marcos-Villar et al., 2009, 2011). Mutation of either the SUMO conjugation site of PML or deletion of SIM within LANA2 significantly impaired PML NB disruption, but the mechanism of this process has remained unclear (Marcos-Villar et al., 2009, 2011). Since LANA2 lacks typical features of a ubiquitin ligase, intrinsic StUbL activity of LANA-2 is unlikely. In addition to the significance of SIM, SUMOylation of LANA2 at multiple K residues are also found to be required for this PML NB disruptive function (Marcos-Villar et al., 2009, 2011). Notably, as LANA2 is a viral latency associated protein, the functional relevance of PML NB disruption by a viral latent protein has not been ascertained experimentally. Altogether, KSHV adopts multiple SUMO-dependent strategies to usurp PML NBs for maintaining latency and further disrupts their structural integrity for rejuvenation and lytic proliferation.

##### Epstein-Barr Virus (EBV)

Epstein-Barr virus, a gammaherpesvirus with a double stranded DNA genome, can cause B cell lymphoma, nasopharyngeal cancer, Hodgkin’s and non-Hodgkin’s lymphomas, and a subset of gastric cancers. It can either be maintained in a latent state and lead to unrestricted cell proliferation and tumorigenesis or can switch to lytic proliferation, especially in the infected epithelial cells. Interestingly, the SUMO system may be involved in maintenance of EBV latent infection. The primary EBV oncoprotein latent membrane protein 1 (LMP1), which is pivotal in modulating viral quiescence, interacts with Ubc9 leading to SUMOylation of many host proteins (Li and Chang, 2003; Bentz et al., 2011). Two important targets of LMP1-mediated SUMOylation are IRF7 and KAP1 (Bentz et al., 2012, 2015). SUMO-conjugated IRF7 at K452 has increased nuclear localization and stability but decreased transactivation potency and therefore is incapable of mounting a robust innate immune response in cells latently infected with EBV (Bentz et al., 2012). SUMOylation of KAP1 increases its transcriptional repression on EBV early promoters and the lytic origin of replication of the virus (Bentz et al., 2015), thereby decreasing transcription of Zta and Rta (EBV lytic trans-activators) and contributing to viral latency (Bentz et al., 2015). None of the LMP1-directed SUMOylation targets resides within PML NB, but LMP1 increases PML expression and immunofluorescence intensity of PML NBs. Disruption of PML NBs results in EBV lytic replication, suggesting that NB disruption is involved in the switch from latent to lytic infection (Sides et al., 2011a, b).

The EBV tegument protein BNRF1 can target PML NBs by interacting with Daxx and disrupting the formation of the Daxx-ATRX Histone H3.3 chaperone complex (Figure 3). Mechanistically, BNRF1 binds to Daxx and outcompetes binding of Daxx to ATRX. Subsequent failure of Histone H3.3 loading onto the viral chromatin leads to enhancement of viral gene expression during primary infection (Tsai et al., 2011, 2014). In addition to preventing Daxx/ATRX/Histone H3.3-mediated heterochromatinization of the viral genome, BNRF1 also facilitates formation of the stabilized ternary complex with Daxx, and histone variants H3.3/H4 and alters the histone dynamics in favor of expression of viral genes at pre-latency stage (Tsai et al., 2014). This further allows expression of latency associated genes and establishment of latent infection.

Disruption of the integrity of PML NBs is crucial for lytic rejuvenation of EBV (Bell et al., 2000). The EBV protein EBNA1 induces degradation of PML through the ubiquitin-proteasome system (Figure 3; Sivachandran et al., 2008). Degradation involves binding to the deubiquitylase USP7 and the kinase casein kinase 2 (CK2), which promotes phosphorylation and subsequent degradation of PML. Disruption of PML NBs also impairs p53 activation, thereby likely contributing to tumorigenesis (Sivachandran et al., 2008, 2010). Interestingly, PML disruption by EBNA1 occurs in epithelial cells, which promote lytic infection, but has not been observed in B lymphocytes, which are predominantly responsible for latent infection, further supporting the idea that dismantling of NBs predisposes latently infected cells to lytic rejuvenation (Sivachandran et al., 2012). Consistently, depleting PML was shown to trigger EBV lytic cycle and supplementing PML depleted cells with single PML isoform (PML I-VI) suppressed EBV lytic reactivation except in case of PML IV which was revealed to be the most vulnerable PML isoform to EBNA1-mediated degradation (Sivachandran et al., 2012).

An immediate-early protein of EBV Zta (BZLF1), which is involved in lytic replication of the virus and transactivation of EBV early genes, can also disrupt PML NBs even under exogenously expressed condition (Figure 3; Adamson and Kenney, 2001). Zta itself can be SUMOylated at K12 (Adamson and Kenney, 2001; Hagemeier et al., 2010; Murata et al., 2010) and it has been proposed that Zta inhibits SUMO conjugation to PML by competing for the limiting pool of endogenous SUMO. Loss of SUMO-modified PML induces the dissolution of PML NBs and a diffuse nuclear distribution of PML (Figure 3; Adamson and Kenney, 2001). Notably, however, partial disruption of PML NBs was still observed upon expression of a SUMOylation-deficient mutant of Zta, implying other viral regulators to have PML NB disruptive functions (Hagemeier et al., 2010). Consistently, another study revealed overexpression of many EBV-encoded proteins such as BDLF1, EBNA3B, BRLF1, BFLF2, BLLF2, and BZLF1 to have the potency to reduce the average number of PML NBs in infected cells (Salsman et al., 2008). Therefore, EBV has developed multiple mechanisms to disintegrate PML NB integrity which emphasizes the significance of these structures during the lytic cycle of this gammaherpesvirus.

There are also reports of viral protein such as the EBV-encoded protein kinase (EBV-PK), and host regulatory protein such as the cellular scaffolding protein RanBPM to dictate Zta SUMOylation status and therefore to influence Zta-mediated lytic reactivation of EBV (Hagemeier et al., 2010; Yang et al., 2015b). Whether these regulatory aspects of Zta SUMOylation have any direct impact on Zta’s ability to disrupt PML NBs has not been addressed yet.

Therefore, in general, herpesviruses exploit the molecular assembly within PML NBs to heterochromatinize their genome and to go into the latent phase of infection. During rejuvenation, they actively disrupt PML NB integrity by specific lytic proteins to trigger lytic proliferation.

#### Adenoviruses

##### Human Adenovirus (HAdV)

In contrast to the early recruitment of herpesviral DNA to PML NBs, adenoviral DNA is associated with PML NBs at a relatively late stage of infection (≥4 h) (Komatsu et al., 2015). Interestingly, this targeting does not require the incoming viral DNA itself but is mediated by the viral single-strand DNA binding protein DBP (also called E2A) which is involved in viral DNA replication (Komatsu et al., 2015). During infection with adenovirus type 5, constituents of PML NBs, including PML and Daxx, are re-organized from their typical punctate appearance to elongated nuclear tracks (Figure 3; Carvalho et al., 1995; Puvion-Dutilleul et al., 1995). Mechanistically, this reorganization involves an interaction of the adenoviral protein E4orf3 with the PML (Figure 3; Doucas et al., 1996; Hoppe et al., 2006). Elegant structural work revealed that E4orf3 forms a multivalent polymer thereby creating avidity-driven interactions with PML and other components of these tracks (Ou et al., 2012). Reorganization of PML NBs fosters viral replication as an E4orf3 mutant that is defective in reorganization is severely growth-compromised. Further, this E4orf3 mutant virus is less potent in antagonizing the IFN-induced antiviral state. Interestingly, however, this phenotype can be rescued upon depletion of PML, underlining the importance of PML in anti-viral defense (Ullman et al., 2007; Ullman and Hearing, 2008). E4orf3-induced nuclear tracks also sequester other antiviral host proteins, such as Mre11 and Nbs1, which are part of the MRN (Mre11-Rad50-Nbs1) complex that mediates the DNA damage response (DDR) and DNA repair (Sohn and Hearing, 2012). The DDR hampers viral DNA replication and HAdV has evolved complementary mechanisms to inhibit the MRN complex. In addition to the sequestration of MRN components within the tracks of infected cell nuclei, the virus mediates ubiquitin-proteasome-dependent degradation of Mre11 through an E3 cullin-RING ligase (CRL) complex assembled by adenoviral proteins E1B-55K in conjunction with E4orf6. Importantly, E4orf3-mediated track sequestration of Mre11 and Nbs1 triggers their SUMOylation potentially facilitating their relocation to cytosolic aggresomes and subsequent degradation (Stracker et al., 2002; Araujo et al., 2005; Liu et al., 2005; Karen et al., 2009; Sohn and Hearing, 2012). Along this line, the transcriptional regulators TIF1γ and TFII-I are also sequestered in PML containing nuclear tracks, where they undergo E4orf3-mediated polySUMOylation and subsequent ubiquitin-proteasom-dependent degradation, most likely through cellular StUbL activity (Bridges et al., 2016; Sohn and Hearing, 2016). Removal of TIF1γ and TFII-I might cause de-repression of specific adenoviral promoters, thereby favoring viral gene transcription. The inactivation of antiviral host factors by virus-induced sequestration or proteasomal degradation is also exemplified by inhibition of Daxx function (Figure 3; Schreiner et al., 2010). In this case it has been proposed that, the adenoviral capsid protein VI interacts with and displaces Daxx from PML NBs to the cytoplasm, thereby de-repressing Daxx-mediated silencing of the viral E1A promoter (Figure 3; Schreiner et al., 2012). Further, SUMO conjugated E1B-55K in conjunction with the cellular StUbL RNF4 was shown to induce proteasomal degradation of Daxx (Endter et al., 2001; Schreiner et al., 2011; Wimmer et al., 2013; Müncheberg et al., 2018). Altogether, these data indicate that reorganization of PML NBs and E4Orf3-mediated formation of tracks in the course of adenovirus infection is a key process in repurposing or inactivating cellular regulators of transcription and DNA repair. Notably, these nuclear tracks also harbor PIAS3 SUMO E3 ligase which might further explain the stimulatory effect of E4orf3 on SUMO conjugation and SUMO chain formation on many host proteins (Higginbotham and O’Shea, 2015; Sohn et al., 2015). Alternatively, E4ORF3 functions as a SUMO E3 ligase and/or E4 chain elongase on selected substrates (Sohn and Hearing, 2016).

Interestingly, the E4orf3-positive tracks reside in close spatial proximity of the adenoviral replication centers (RCs) and a recent report has unveiled the pivotal role of SUMOylation for this positioning (Stubbe et al., 2020). In particular, E2A was found to undergo SUMO conjugation, thereby fostering interaction with both PML and Sp100A and convergence of PML NB tracks with the adenoviral RCs (Figure 3). A SUMOylation-deficient E2A mutant exhibits reduced binding to PML and Sp100A, and accordingly, viral RCs and PML tracks remain separated. Compared to their wild type counterpart these mutant viruses are less efficient in yielding progeny virions (Stubbe et al., 2020). Juxtaposition of viral RCs to PML NBs may promote adenoviral early gene transcription through PML-mediated recruitment of the viral E1A-13S protein (Figure 3; Berscheminski et al., 2013). The compartmentalization of PML NB components within the adenoviral RCs or E4orf3-containing tracks is a very selective process that is, at least partially, regulated by SUMO-dependent mechanisms (Berscheminski et al., 2014). Sp100B, Sp100C, and Sp100HMG are delocalized to the adenoviral RCs possibly as part of a viral strategy to counteract the restrictive functions of these specific Sp100 isoforms on chromatin architecture. Sp100A, on the other hand, is retained around the periphery of the RCs as a part of the non-canonical track-like PML NB structures, where it favors transcriptional activation of the adenoviral genome (Berscheminski et al., 2014). Mechanistically, adenoviral infection triggers loss of SUMO2 conjugated Sp100A leading to reduced association between Sp100A and HP1, explaining chromatin decondensation by Sp100A on adenoviral promoters (Berscheminski et al., 2014).

Apart from E4orf3, E1B-55K also exploits the SUMO system to target host cell proteins. E1B-55K itself is modified by SUMO and the modification facilitates its nuclear retention by blocking a CRM-dependent nuclear export signal (Kindsmüller et al., 2007; Wimmer et al., 2013). Further, SUMOylation of E1B-55K is important for its interaction with PML IV (Figure 3; Wimmer et al., 2010). Interestingly, E1B-55K can also induce SUMOylation of several PML NB-associated host proteins, including p53 and Sp100. Inhibition of p53 activity by E1B-55K is a hallmark of HAdV infection and E1B-55K appears to apply different strategies to inhibit the antiproliferative and pro-apoptotic activity of p53. One strategy is the ubiquitin-proteasome-mediated degradation of p53 by the above-mentioned CRL/E1B-55K/E4orf6 ubiquitin ligase complex (Figure 3; Querido et al., 2001). Intriguingly, E1B-55K also induces SUMOylation of p53 thereby rendering it transcriptionally inactive by facilitating its nuclear export through transient interactions with PML NBs (Figures 3, 4; Muller and Dobner, 2008; Pennella et al., 2010). Whether E1B-55K exerts E3 SUMO ligase activity by its own or stimulates SUMOylation by co-operating with host E3 ligases is a matter of debate (Muller and Dobner, 2008; Pennella et al., 2010). In addition to p53, E1B-55K also triggers SUMOylation of Sp100A and sequesters it within PML containing nuclear tracks (Figures 3, 4), thereby limiting stimulatory effects of Sp100A on transactivation potential of p53 (Berscheminski et al., 2016). Moreover, ATRX is targeted by the CRL/E1B-55K/E4orf6 complex for proteasomal degradation (Figure 3; Schreiner et al., 2013).

The adenoviral protein E1A also has PML NB destabilizing properties (Figure 3). The mechanism involves a direct interaction between the conserved region 2 of E1A and the N-terminal region of Ubc9 which binds SUMO and regulates polySUMOylation (Figures 3, 4; Yousef et al., 2010). Therefore, interaction of Ubc9 with E1A might affect polySUMOylation, which might further destabilize PML NB integrity (Figure 3; Yousef et al., 2010). At least in a polySUMOylation assay in yeast, wild-type E1A, but not a mutant defective in Ubc9 binding, inhibited polySUMOylation. Moreover, the authors also speculated that E1A binding might affect the ability of Ubc9 to be SUMOylated at K14 residue. As SUMO modification at this residue affects Ubc9 substrate specificity, this interaction might also alter SUMOylation of host proteins in general (Yousef et al., 2010).

Therefore, HAdV selectively usurps certain components of PML NB components at the expense of antagonizing others to favor its own perpetuation.

##### Chicken Embryo Lethal Orphan (CELO) Adenovirus

Chicken embryo lethal orphan adenovirus adopts a robust SUMO-dependent mechanism of PML NB disruption. The viral early protein Gam1 induces ubiquitin-proteasome-dependent degradation of the dimeric SUMO activating enzyme SAE1/SAE2 (Figure 4) by recruiting a cullin-RING E3 ubiquitin ligase (Cul2/5- Rbx1-Elongin B/C) complex (Boggio et al., 2004, 2007). The resulting loss of SUMO conjugation is transduced to the disruption of PML NBs possibly as a result of absence of PML SUMOylation (Colombo et al., 2002; Boggio et al., 2007). As SUMOylation usually leads to transcriptional repression, this global loss of SUMOylation might have a pro-viral significance (Boggio et al., 2004).

#### Human Papillomavirus (HPV)

Human papillomavirus, belonging to the *Papillomaviridae* family, possess limited arsenal to counteract host immune responses because of their small genome size and therefore employ a fundamentally different strategy to manipulate with the PML NBs. Unlike other viruses where PML NBs are associated with restrictive chromatin environment and curtailment of viral lytic gene expression and lytic proliferation, HPVs exploit PML NBs for replication. Accordingly, HPV pseudoviruses are more potent in infecting PML expressing cells than PML depleted cells (Day et al., 2004). Consistently, in absence of PML, transcript and genome levels of bovine papillomavirus 1 (BPV1) were greatly reduced. Moreover, depletion of PML in HaCaT cells triggered significant reduction of both viral transcripts and nuclear load of viral genomes. Interestingly, co-inhibition of JAK/STAT signaling restored the loss of viral genomes, but not viral transcription, suggesting possible antiviral implications of IFN-mediated immune response in absence of PML. In consistence with this, PML depleted HeLa cells, which are intrinsically transformed with HPV 18 oncogenes E6 and E7 and therefore might sustain a perturbed JAK/STAT response, were found to be as supportive as their wild type counterpart in sustaining HPV infection (Hong et al., 2011; Reiser et al., 2011; Bienkowska-Haba et al., 2017). Altogether, PML may provide a protective environment for the HPV genome against innate immune recognition and subsequent degradation and also favors HPV transcription. Agreeably, HPV 16 pseudogenomes colocalized with PML protein in both HaCaT and HeLa cells (Bienkowska-Haba et al., 2017).

Unlike other viruses that disrupt PML NBs, HPVs usurp molecular condensates of PML upon their reformation after mitosis. Mitotic entry coincides with PML deSUMOylation and PML NB disintegration. Subsequently, cytoplasmic PML aggregates, called mitotic accumulations of PML proteins (MAPPs), are formed by homo-multimerization of PML proteins through the RBCC/TRIM motif. These cytosolic PML condensates which even persist in the cytosol after completion of mitosis and nuclear envelope reformation, lack typical PML NB components. When PML translocates back to the daughter cell nuclei, canonical nuclear PML NBs comprising of SUMOylated PML and NB clients are reassembled (Everett et al., 1999; Dellaire et al., 2006). Notably, the HPV genome which remains within an uncharacterized vesicular compartment along with the major viral capsid protein L1 requires disassembly of the nuclear membrane during mitosis to get delivered within the nucleus (Pyeon et al., 2009; Aydin et al., 2014; DiGiuseppe et al., 2016; DiGiuseppe et al., 2017). Another capsid protein L2 has a transmembrane localization and the extra-membrane domain of L2 mediates transport of the genome-containing vesicle along mitotic microtubules toward the condensed chromosomes (DiGiuseppe et al., 2016; Aydin et al., 2017; Day et al., 2019). The viral genome remains in the transport vesicle shortly after completion of mitosis and nuclear envelope reformation (DiGiuseppe et al., 2016; Day et al., 2019). HPV has been shown to interact with PML NBs through L2 after nuclear delivery as part of the viral life cycle (Figure 3). L2 of BPV 1 was initially identified to localize to PML NBs (Day et al., 1998). Subsequent studies also detected HPV 33 L2 protein at PML NBs and reported displacement of Sp100, but not of PML, from these structures (Florin et al., 2002; Becker et al., 2003). Moreover, HPV 16 L2 protein and BPV 1 pseudogenomes were also found to localize to PML NBs (Day et al., 2004). In PML depleted cells, L2 protein shows diffused pattern in the nucleus.

The mechanism of how HPV L2 protein fosters association of the HPV genome with PML NBs is still poorly understood, but SUMO-dependent regulation appears to be involved in this process. L2 protein itself contains one SUMO conjugation motif at K35 which was evidenced to modulate viral capsid assembly (Marusic et al., 2010), but its contribution to interact with PML has not been investigated yet. L2 also has one highly conserved SIM and two additional putative SIMs (Bund et al., 2014). The conserved SIM has been experimentally shown to be important for interaction with PML as mutation of the SIM prevented localization to PML NBs and reduced infectivity independent of the L2 SUMOylation status. A recent report shed light on the nature of this recruitment where PML protein was found to get recruited to the viral genomes and assemble around them, rather than the viral genomes targeting preformed PML NBs (Guion et al., 2019). This engulfment temporally coincides with post-mitotic early interphase while PML protein is translocating back to the nucleus. Spatially, too, this entrapment is unique as PML protein co-localizes with viral genomes that are still residing within transport vesicles. This observation suggests that viral genomes are continuously protected from cellular sensors, first by the transport vesicle, then by PML protein which may provide a protective environment for the genome in the nucleus of host cells to prevent innate immune recognition (Guion et al., 2019). It seemed also plausible that HPV usurps the dissociation of PML NB during mitosis to modify the composition of PML NB rather than reorganizing preassembled structures (Guion et al., 2019; Guion and Sapp, 2020).

Interestingly, unlike PML, Sp100 has been observed to act as a restriction factor for HPV transcription and replication but to be unimportant for the maintenance phase of the HPV life cycle (Stepp et al., 2013, 2017). Consistently, viral transcription and replication from transfected HPV 18 genome in primary human keratinocytes were greatly enhanced under the condition of Sp100 depletion (Stepp et al., 2013). Also in line with this is the observation that recruitment of Sp100 to the HPV genome occurs at late interphase and in a temporally delayed manner compared to PML recruitment (Guion et al., 2019). Furthermore, Sp100 was only observed to co-localize with viral genomes after the loss of the transport vesicles and only when PML protein had already been recruited. A justification has been put forward that the delayed recruitment of Sp100 perhaps allows a window where a burst of initial viral transcription ensues in absence of Sp100 before Sp100-mediated repression of viral transcription sets in to promote the skewing of viral life cycle toward the maintenance phase (Guion et al., 2019). Of note, knocking down Daxx expression did not have an impact on the HPV18 transcription and replication implying dispensability of Daxx in this process (Stepp et al., 2013).

### RNA Viruses

#### Picornaviruses

##### Encephalomyocarditis Virus (EMCV)

Though not as elaborately studied as DNA viruses, there are examples of RNA viruses interacting with the host cellular SUMOylation machinery and PML NBs. One classical example includes anti-PML activities of encephalomyocarditis virus (EMCV) protease 3C. EMCV which belongs to the family *Picornaviridae* can cause neurological and reproductive complicacies in many mammals, likely including humans through zoonosis. Interestingly, primary fibroblasts derived from PML knockout mice are more vulnerable to infection with EMCV, suggesting that the PML protein might act as a restriction factor for EMCV replication (El McHichi et al., 2010). Moreover, progressive infection with EMCV was found to cause a reduction in PML protein levels both in IFN-treated cells and in PML III-expressing cells. The degradation event was preceded by translocation of PML from nucleoplasm to the nuclear matrix and SUMO1, SUMO2/3 conjugation to PML, leading to an increase in PML NB size. Subsequently, the viral protease 3C was found to localize to the PML NBs along with proteasome components to trigger PML degradation in a proteasome- and SUMO-dependent manner (Figure 3); PML SIM, however, proved to be dispensable for the degradation (El McHichi et al., 2010). Unlike PML III which is antagonized by EMCV protease 3C, SUMO-conjugated PML isoform IV has been reported to interact with the EMCV 3D polymerase via the isoform’s unique C-terminal domain and to sequester the viral protein to PML NBs (Figure 3), thereby impairing viral perpetuation (Maroui et al., 2011). Consistently, selective depletion of PML IV also undermines the anti-EMCV potency of IFN (Maroui et al., 2011).

##### Poliovirus

SUMOylation of PML has been reported to have indirect effects on the life cycle of poliovirus, another *Picornaviridae* family member etiologically responsible for paralytic poliomyelitis. The mechanism includes virus-mediated activation of the extracellular-signal-regulated kinase (ERK), which phosphorylates PML, thereby facilitating PML SUMOylation and translocation to the nuclear matrix. Subsequently, p53 gets recruited to PML NBs and becomes phosphorylated on Ser15 in a PML-dependent way (Figure 3; Pampin et al., 2006). Therefore, poliovirus infection results in PML-dependent p53 activation and mobilization of apoptotic cascade downstream of activated p53, resulting in inhibition of viral replication. PML III overexpression conferred resistance to poliovirus in p53 wild-type cells, but not in p53-inactive cells, while depletion of PML III abolishes p53-dependent apoptosis and antiviral effects. Interestingly, as a countermeasure against this transient cyto-protective effects, poliovirus induces degradation of p53 through proteasome via the E3 ubiquitin ligase Mdm2 (Pampin et al., 2006).

#### Rhabdoviruses

##### Vesicular Stomatitis Virus (VSV)

Antiviral effects of PML have also been extended to VSV, a member of the *Rhabdoviridae* family which infects human zoonotically, as exogenous PML III conferred resistance to VSV by inhibiting viral mRNA and protein synthesis (Chelbi-Alix et al., 1998), while PML knockout mice were found to be more susceptible to VSV infection than wild type mice (Bonilla et al., 2002). Disruption of PML NBs, however, has not been observed in VSV infected cells. The mechanism of how PML exerts antagonistic activity against a virus, whose replication takes place entirely in the cytoplasm and which does not disrupt PML NBs, is yet to be addressed. The observation that only the coiled-coil domain deletion mutant of PML, but not the other RING finger PML mutant and cytoplasmic PML III mutant, fails to show the antiviral activity, emphasizes the importance of the coiled-coil region of PML for anti-VSV effects (Chelbi-Alix et al., 1998). Another report has also elucidated PML IV to have anti-VSV activity (El Asmi et al., 2014). Mechanistic profiling elucidated PML IV to potentiate both the intrinsic and innate immune arms of the antiviral defense pathway. The innate immune response involves SUMOylated PML IV to interact with and recruit peptidyl-prolyl cis/trans isomerase (Pin1) to PML NBs. This results in activated IRF3 to escape from Pin1-mediated degradation and to potentiate its canonical functions of IFN-β synthesis. Interestingly, though depletion of IFR3 abrogated PML IV-induced IFN synthesis, it could not entirely inhibit PML IV-induced inhibition of viral proteins, suggesting parallel IFN-dependent and independent pathways to operate downstream of PML IV. Moreover, the agonistic effects of SUMOylated PML IV to stimulate IFN-β synthesis was found to be a broader effect on innate immune regulation rather than a VSV infection-specific scenario (El Asmi et al., 2014).

##### Rabies Virus

Unlike VSV, another *Rhabdoviridae* family member rabies virus which causes central nervous system dysfunctions does reorganize PML NBs during infection to form larger and more electron-dense aggregates. The P3 protein, an amino-terminally truncated product of the viral P mRNA which also codes for phosphoprotein P, causes this effect. Interestingly, sole expression of P delocalizes PML III from nuclear to cytoplasmic puncta where both proteins colocalize (Figure 3). A direct interaction was also evidenced between the C-terminal domain of P protein and the PML RING finger. However, the observations that overexpression of PML III isoform does not perturb rabies infection but that PML^–^/^–^ MEFs are more susceptible to rabies infection than wild-type MEFs, do suggest involvement of other PML isoforms to take part in the anti-rabies defense (Blondel et al., 2002). Indeed, subsequent studies revealed the antiviral importance of PML IV, but not of other nuclear as well as cytoplasmic PML isoforms, to act as a restriction factor against rabies infection independent of IFN signaling axis. Notably, PML IV SUMO conjugation was found to be essential for its antiviral effect as the protective effect of PML IV was lost when the SUMOylation sites were mutated (Blondel et al., 2010).

#### Arenavirus

##### Lymphocytic Choriomeningitis Virus (LCMV)

The RING finger protein Z of LCMV, an *Arenaviridae* family member which transmits zoonotically to human leading to aseptic meningitis, induces redistribution of PML from nuclear condensates to cytoplasmic aggregates during infection (Figure 3). In these aggregates, both proteins interact with the translation initiation factor eukaryotic initiation factor 4E (eIF-4E) and reduce eIF-4E’s affinity for the 5′ mRNA cap structure, thereby inhibiting cellular translation (Borden et al., 1998; Kentsis et al., 2001). Subsequently, PML has been shown to act as a restriction factor during infection with LCMV as PML knockout mice showed more susceptibility to LCMV infection than their wild type counterparts (Bonilla et al., 2002). Consistently, anti-LCMV potency of IFN was also found to be partially sensitive to PML knocking out in MEFs, suggesting PML to be one of the IFN-induced antiviral mediators for defense against LCMV (Djavani et al., 2001). However, neither overexpression of PML III nor Sp100 had any repressive effect on LCMV growth (Asper et al., 2004), implicating PML isoforms other than PML III to have anti-LCMV relevance.

#### Orthomyxovirus

##### Influenza Virus (IFV)

Like other RNA viruses, PML exerts antagonistic effects during infection with the *Orthomyxoviridae* family member influenza A virus in a viral strain-specific manner (Li et al., 2009). Overexpression of different PML isoforms such as PML III, PML IV, and PML VI attenuates influenza A viral propagation in cell culture (Chelbi-Alix et al., 1998; Iki et al., 2005; Li et al., 2009), while PML silencing has an agonistic effect (Iki et al., 2005; Li et al., 2009). However, neither the mechanism by which PML exerts anti-influenza activity nor the significance of PML NB recruitment of viral proteins (the matrix protein M1 and the nonstructural polypeptides NS1 and NS2) (Iki et al., 2005) has been addressed yet. A more recent study suggested induction of a specific host cellular SUMOylation response in influenza virus infected cells. However, PML SUMOylation was insensitive to this infection. Interestingly, confocal imaging showed PML-stained puncta to get smaller and more in number whereas Sp100 and Daxx appeared dispersed in the nuclear compartment of infected cells, suggestive of PML NB dispersion (Domingues et al., 2015).

#### Flaviviruses

##### Hepatitis C Virus (HCV)

Interplay of HCV, a member of the family *Flaviviridae*, with the host cellular PML NBs involves the HCV core protein to get recruited to PML NBs by interacting with PML IV and to inhibit the co-activator activity of PML IV on p53 (Figure 3). This leads to prevention of p53-dependent apoptotic induction and development of HCV-associated hepatocellular carcinoma (Herzer et al., 2005). However, SUMO-dependent regulation of PML NB structures has not yet been reported for HCV.

##### Dengue Virus (DENV)

Promyelocytic leukemia has recently been identified as an antiviral host cellular determinant capable of restricting all serotypes of DENV, another *Flaviviridae* family member, as siRNA-mediated depletion of PML resulted in increased virus production (Giovannoni et al., 2015, 2019). Moreover, as a part of viral countermeasures, PML NBs were found to get significantly reduced in number during infection with DENV serotypes. The causative agent was identified to be the non-structural viral protein NS5 which co-localized with PML III and IV at PML NBs and promoted accelerated degradation of PML III and IV (Figure 3; Giovannoni et al., 2019). Consistently, over expression of these specific PML isoforms curtailed DENV replication and progeny yield. Of note, Sp100 and Daxx were proved to be dispensable for DENV progeny production, suggesting specific anti-DENV implications of PML III and IV (Giovannoni et al., 2019). Interestingly, DENV NS5 interacts with Ubc9 and undergoes SUMOylation. This SUMOylation requires a SIM domain within NS5 (Su et al., 2016). Any correlation of SUMO conjugation of NS5 on its PML NB disruptive property, however, is yet to be addressed experimentally.

#### Retroviruses

##### Human Immunodeficiency Virus-1 (HIV-1)

During infection with HIV-1, a lentivirus belonging to the family *Retroviridae*, PML NBs rapidly relocalize from nuclear to cytoplasmic aggregates (Figure 3). Further, PML depletion increased reverse transcription capacity of HIV-1. However, involvement of PML is likely indirect through stabilization of Daxx which sensitized the reverse transcription of HIV-1 RNA by spatially relocating to incoming viral capsids in the cytoplasm. Similar relocation of PML NB components and Daxx-dependent antagonistic impact of PML were also observed in other retroviruses such as simian immunodeficiency virus (SIVmac), murine leukemia virus Moloney and equine infectious anemia virus. Interestingly, SUMOylation inhibitor ginkgolic acid (that impairs the formation of the E1-SUMO intermediates) strongly sensitized formation of cytoplasmic PML-containing aggregates in HIV-1 infected cells, suggesting importance of SUMO conjugation for structural integrity of these cytoplasmic aggregates. However, data based on ginkgolic acid treatment need to be interpreted with a note of caution since it is a rather unspecific SUMO inhibitor. Further, no other specific SUMO target specifically directing this delocalization has been assigned (Dutrieux et al., 2015). An interesting recent report also elucidated another SUMO-dependent PML-NB disruptive strategy by the HIV-1 accessory protein Vpu. In brief, Vpu interacts with RanBP2 at nuclear pore to inhibit the SUMO E3 ligase activity of the RanBP2–RanGAP1^∗^SUMO1–Ubc9 complex, thereby affecting the PML NB integrity (Figure 3) and SUMOylation of DNA repair proteins BLM, Rad52. As a consequence, Rad52-dependent homologous repair and circularization of non-integrated linear DNA from a superinfecting virus were prevented. Moreover, BLM-dependent nucleolytic attack on those susceptible DNA ends were facilitated (Volcic et al., 2020). On a different note, PML aggregation was observed in syncytia present in the brain or lymph nodes of infected patients or in syncytia elicited by the envelope glycoprotein complex of HIV-1 *in vitro* (Perfettini et al., 2009). PML was shown to foster phosphorylation of ATM which was co-recruited with PML in nuclear aggregates and mobilized p53-dependent syncytial apoptosis in HIV-infected CD4 cells (Perfettini et al., 2009). However, any mechanistic role of SUMO-mediated regulation has not been addressed in this respect.

##### Human Foamy Virus (HFV)

Human foamy virus are retroviruses infecting non-human primates but can be transmitted to human through zoonotic infection. Though endogenous PML has been shown not to interfere with HFV replication and latency and also not to get delocalized during infection (Regad et al., 2001; Meiering and Linial, 2003), PMLIII overexpression reportedly attenuates HFV transcription by interacting with the HFV transactivator protein Tas and hindering its binding to DNA (long-terminal repeat and internal promoter) (Regad et al., 2001). However, this interaction was revealed to be independent of SUMO regulation. Anti-viral potency of IFN is also diminished in PML deficient MEFs but other IFN-dependent antiviral effectors such as MxA, Mx1, and PKR could not sensitize HFV replication (Regad et al., 2001), again bolstering the specificity of PML in creating an IFN-induced antiviral state against HFV.

### Bacterial Pathogens

Alteration in the SUMOylation landscape is also reported in response to bacterial infection, indicating a broader implication of SUMO proteins in controlling infection and immunity. PML knockout mice have been found to be more sensitive to infection by *Listeria monocytogenes*, a Gram-positive bacterium that is responsible for the foodborne disease listeriosis (Lunardi et al., 2011). A subsequent study revealed that SUMOylation status of PML largely serves as a sensor of *Listeria* infection (Ribet et al., 2017). The pore-forming toxin listeriolysin O (LLO) was found to be the determining factor for such response (Ribet et al., 2017). One of the multi-faceted signaling events that ensues upon LLO-mediated perforation of the infected host cell membrane is the proteasome-independent degradation of Ubc9 (Figures 3, 4; Ribet et al., 2010). Impairment of *de novo* SUMOylation coupled to unrestricted activity of SUMO proteases results in massively deSUMOylated population of PML (Figure 3) which gets multimerized and associated with the nuclear matrix in response to infection-induced oxidative stress. This PML deSUMOylation event has been shown to dampen bacterial multiplication. Functionally potent HCMV IE1 protein which is capable of triggering PML deSUMOylation was shown to substitute LLO action and reduced bacterial replication efficiency. Notably, in addition to PML, Sp100 deSUMOylation was also distinct in presence of LLO (Ribet et al., 2017). Antibacterial activity of PML can also be attributable to a number of immunity and cytokine genes that are transcriptionally regulated by PML independent of LLO (Ribet et al., 2017). In contrast to PML, deSUMOylation of many cellular factors, especially transcription factors, has been shown to be beneficial for the bacteria by favoring bacterial replication or survival in the host cells (Impens et al., 2014). One of such candidates is the SUMO-stabilized SMAD4 which acts as a transactivator during TGFβ signaling. LLO was found to destabilize SMAD4 through deSUMOylation leading to impairment of the cyto-protective TGFβ signaling (Ribet et al., 2010). These results suggest a finely tuned SUMO response that occurs during *Listeria* infection and controls the bacterial pathogenesis. Of note, similar to LLO, other bacterial pore-forming toxins [perfringolysin O (PFO; from *Clostridium perfringens*) and pneumolysin (PLY; from *Streptococcus pneumoniae*)] were also evidenced to induce PML deSUMOylation and multimerization (Figure 3; Ribet et al., 2017). Moreover, in line with the observation that membrane association and the pore-forming activity of LLO are essential for Ubc9 degradation, PFO and PLY show a comparable reduction in cellular levels of Ubc9 (Figures 3, 4; Ribet et al., 2010). Strikingly, however, PML was found to have no role in controlling infection by *Salmonella typhimurium*, a bacterium that is responsible for gastroenteritis in humans, even though this pathogen was also shown to induce a decrease in Ubc9 level leading to inhibition of host cellular SUMOylation (Figure 4; Decque et al., 2016; Ribet et al., 2017). The reduction in Ubc9 level is due to induction of two microRNAs, miR30c and miR30e, which specifically target Ubc9 within infected host cells (Verma et al., 2015). In case of *Shigella flexneri*, which is another gastroenteritis-causing bacterium, infection is associated with an influx of calcium into the host cell. This ion flux activates the host calpain proteases which cleave SAE2, one of the two components of the E1 SUMO enzyme (Figure 4; Lapaquette et al., 2017). The resulting inhibition of SUMOylation is associated with increased *Shigella* entry (Fritah et al., 2014; Lapaquette et al., 2017). Moreover, another mechanism of global impairment of SUMOylated proteins (both SUMO1 and SUMO2/3 conjugated) in *Shigella* infected cells was revealed to be mediated by the bacterial type 3 Secretion System (T3SS) which triggered a proteasome-dependent Ubc9 destabilization (Figures 3, 4). Whether proteasomal degradation is preceded by proteolytic ubiquitination is, however, not known (Sidik et al., 2015). Not surprisingly, therefore, overexpression of components of the SUMO pathway significantly decreased the ability of *Shigella* to enter cultured epithelial cells. Corroborative *in vivo* data in an infection model of haploinsufficient Ubc9^+^/^–^ mice also showed profound destruction of colonic tissue, increased intestinal permeability, and increased signatures of pro-inflammatory cytokines in comparison to wild type controls, suggesting SUMO response to curtail *Shigella* infection at multiple steps (Fritah et al., 2014). Indeed, quantitative proteomic studies revealed *Shigella* infection to be associated with the SUMO-2 modification of multiple transcriptional regulators which control inflammatory response (Fritah et al., 2014).

Notably, the number of PML NBs increases during infection with *Shigella* (Sidik et al., 2015). However, this increase is not accompanied by the enhanced expression of endogenous PML, suggesting the occurrence of PML NB fission or *de novo* PML body nucleation from soluble PML. *Shigella* infection also triggered alteration in the SUMO1 localization and abundance. In cells infected with wild-type *Shigella*, SUMO1 was visualized as more condensed puncta with sharper edges and brighter staining in comparison to cells that were uninfected or infected with non-invasive *Shigella*. However, no correlation was found between PML body number and the condensed SUMO1 phenotype (Sidik et al., 2015). Thus, many extracellular pathogens presumably target the SUMO modification system to manipulate with the PML NBs, thereby arguing for an evolutionarily conserved strategy in some pathogenic bacteria to increase infectivity by restricting SUMO-dependent host cell responses.

## Conclusion and Perspectives

Promyelocytic leukemia NBs have been emerging to be sub-nuclear niches where SUMO-SIM interactions guide the assembly of a heterogeneous macromolecular complex. The formation involves liquid-liquid phase separation in matrix-associated spherical condensates but allows a dynamic equilibrium with the surrounding nucleoplasmic contents. With the growing list of client proteins which are shown to be transiently associated with PML NBs (Van Damme et al., 2010), functional implications of these sub-nuclear foci are expanding. PML NBs have been traditionally regarded as hubs of intrinsic immune defenses against invading pathogens. In light of some current discoveries, however, a new dimension has emerged indicating components of PML NBs to be differentially regulated during infection with some pathogens such as HAdV and HPV. Consistently, certain PML NB components have been revealed to have anti-pathogenic functions whereas others have shown to foster progression of infection for the same pathogen. This reiterates the classical pathogen-mediated host cellular reprogramming where host determinants are more often the specific targets of manipulation rather than stochastic off targets. From a different perspective, PML NB-mediated repression of gene expression has been proved to be pivotal for triggering and/or maintaining latency in many viral infections, suggesting pro-viral implications of these host determinants in establishing prolonged viral persistence. Of note, many viral proteins which play critical roles in dictating PML NB physiology are now emerging to be the functional (and sometimes, even structural) counterparts of the cellular SUMO machinery such as SUMO E3 ligases, SENPs or StUbLs, suggesting implications of the host cellular SUMO modulations on viral evolutionary dynamics. With the recent advances and refinements at the technological frontier, future studies will likely unveil many uncharacterized SUMO-dependent PML NB regulations in the context of pathogen infected cells. Apart from their obvious scholarly significance, these endeavors will also propel research focused on therapeutic importance. Indeed, Cambogin, a targeted small molecule inhibiting the SIM within KSHV LANA protein has been proved to have anti-KSHV function at sub-cytotoxic dose (Ding et al., 2019). Moreover, in coherence with the agonistic effects of PML in governing innate immune response, novel SUMO-dependent intersections between PML NBs and pathogenic organisms will be of significant relevance in future.

## Author Contributions

UP and SM wrote the manuscript. UP prepared the figures. Both authors approved the final version of the manuscript.

## Conflict of Interest

The authors declare that the research was conducted in the absence of any commercial or financial relationships that could be construed as a potential conflict of interest.

## Publisher’s Note

All claims expressed in this article are solely those of the authors and do not necessarily represent those of their affiliated organizations, or those of the publisher, the editors and the reviewers. Any product that may be evaluated in this article, or claim that may be made by its manufacturer, is not guaranteed or endorsed by the publisher.
